# Chemical-proteomics Identify Peroxiredoxin-1 as an Actionable Target in Triple-negative Breast Cancer

**DOI:** 10.7150/ijbs.78554

**Published:** 2023-03-13

**Authors:** Elena Spínola-Lasso, Juan Carlos Montero, Roberto Jiménez-Monzón, Francisco Estévez, José Quintana, Borja Guerra, Khaled M. Elokely, Francisco León, Henoc del Rosario, Leandro Fernández-Pérez, Manuel Rodríguez López, Bonifacio Nicolás Díaz-Chico, Grant McNaughton-Smith, Atanasio Pandiella, Juan Carlos Díaz-Chico

**Affiliations:** 1 Instituto Universitario de Investigaciones Biomédicas y Sanitarias (IUIBS), Departamento de Bioquímica y Biología Molecular, Fisiología, Genética e Inmunología, Universidad de Las Palmas de Gran Canaria, The Canary Islands, Spain.; 2 Institute of Biomedical Research of Salamanca (IBSAL), Instituto de Biología Molecular y Celular del Cáncer-CSIC and CIBERONC, Salamanca, Spain.; 3 Instituto Canario de Investigación del Cáncer (ICIC), The Canary Islands, Spain.; 4 Instituto Universitario de Investigaciones Biomédicas y Sanitarias (IUIBS), Farmacología Molecular y Traslacional, Departamento de Ciencias Clínicas, Universidad de Las Palmas de Gran Canaria, The Canary Islands, Spain.; 5 Unidad de Biomedicina asociada al CSIC, Instituto Universitario de Investigaciones Biomédicas y Sanitarias (IUIBS), Universidad de Las Palmas de Gran Canaria, The Canary Islands, Spain and Instituto de Investigaciones Biomédicas “Alberto Sols'' CSIC - Universidad Autónoma de Madrid, Madrid, Spain; 6 Institute for Computational Molecular Science and Department of Chemistry, Temple University, Philadelphia, USA.; 7 Department of Drug Discovery and Biomedical Sciences, College of Pharmacy, University of South Carolina, Columbia, USA.; 8 Centro Atlántico del Medicamento S.A. (CEAMED S.A), La Laguna, The Canary Islands, Spain.

**Keywords:** triple-negative breast cancer, quinone-fused oxazepine, peroxiredoxin-1, oxidative stress

## Abstract

Triple-negative breast cancer (TNBC) is difficult to treat; therefore, the development of drugs directed against its oncogenic vulnerabilities is a desirable goal. Herein, we report the antitumor effects of CM728, a novel quinone-fused oxazepine, against this malignancy. CM728 potently inhibited TNBC cell viability and decreased the growth of MDA-MB-231-induced orthotopic tumors. Furthermore, CM728 exerted a strong synergistic antiproliferative effect with docetaxel *in vitro* and this combination was more effective than the individual treatments *in vivo*. Chemical proteomic approaches revealed that CM728 bound to peroxiredoxin-1 (Prdx1), thereby inducing its oxidation. Molecular docking corroborated these findings. CM728 induced oxidative stress and a multi-signal response, including JNK/p38 MAPK activation and STAT3 inhibition. Interestingly, Prdx1 downregulation mimicked these effects. Finally, CM728 led to DNA damage, cell cycle blockage at the S and G_2_/M phases, and the activation of caspase-dependent apoptosis. Taken together, our results identify a novel compound with antitumoral properties against TNBC. In addition, we describe the mechanism of action of this drug and provide a rationale for the use of Prdx1 inhibitors, such as CM728, alone or in combination with other drugs, for the treatment of TNBC.

## Introduction

Breast cancer is the most commonly diagnosed malignancy in women and one of the leading causes of cancer-related deaths 1. Breast cancer is a complex heterogeneous disease that is clinicopathologically classified into hormone-receptor-positive, human epidermal growth factor receptor 2 (HER2) positive, and triple-negative breast cancer (TNBC) subtypes. TNBCs lack the expression of estrogen and progesterone receptors, and do not overexpress HER2. TNBCs account for 15-20% of all diagnosed breast cancers and occur more frequently in younger women, those of African and Hispanic heritage, and those with harmful germline mutations in breast cancer susceptibility genes 2.

Unlike hormonal and HER2 positive breast cancers, there is a lack of known oncogenic driver alterations in TNBC. This has hampered the development of target-specific therapies 3. Despite these inconveniences, three types of targeted drugs have reached the TNBC clinics in recent years. One of these is the use of poly (ADP-ribose) polymerase (PARP) inhibitors 4. These drugs are effective in a subset of patients whose tumors have inactivating mutations in breast cancer genes (BRCA). Second, antibody-guided topoisomerase inhibitors such as sacituzumab govitecan 5. The third group includes immune checkpoints inhibitors that target the programmed cell death protein 1/programmed cell death ligand 1 (PD-1/PD-L1) axis 6. These novel treatments have been combined with classical treatments, mainly those based on chemotherapeutic agents 7. Chemotherapy-based regimens in combination with surgery or radiotherapy are the most widely accepted options for TNBC patients, although their effectiveness is limited 8, 9. Despite these advances in disease management, TNBC remains incurable at advanced stages. Therefore, the identification of novel drugs targeting TNBC vulnerabilities is a desired goal.

The antineoplastic activities of benzoxazepine and naphthoquinone derivatives have been widely recognized. The anticancer effects of benzoxazepine derivatives are mediated by several mechanisms, including phosphoinositide 3-kinase (PI3K) inhibition 10, interruption of tubulin dynamics 11, cyclin dependent kinase 8 (CDK8) inhibition 12, and Janus kinase/signal transducer and activator of transcription 3 (JAK/STAT3) signaling inhibition 13. Taselisib is an indole-fused benzoxazepine that has been tested in combination with an estrogen receptor antagonist in phase III clinical trials for estrogen receptor (ER) positive and HER2 negative advanced breast cancer patients with mutated PIK3CA 14. On the other hand, the action of 1,4-naphthoquinones (1,4-NQ) is mainly associated with two chemical properties: their ability to interact with nucleophilic targets by reversible or non-reversible covalently binding, and their capability of generating reactive oxygen species (ROS) 15 - 17. Accordingly, the anticancer effect of 1,4-NQ is mediated by multiple molecular mechanisms including DNA damage, inhibition of STAT3 and topoisomerase II, and regulation of the tumor suppressor factor p53 16, 17. Napabucasin is a furo-1,4-NQ derivative that has been tested in combination with other chemotherapeutic drugs in phase III clinical trials for metastatic pancreatic carcinoma 18 and refractory advanced colorectal cancer 19. The antineoplastic action of taselisib and napabucasin opens up the possibility of creating chimeric chemicals based on the backbone structures of these compounds, with the final aim of combining their antitumor properties.

In this study, we generated a novel naphthoquinone-fused benzoxazepine (CM728). We found that CM728 exerted an antitumor effect on TNBC either alone or in combination with docetaxel. Using chemical proteomic approaches, we identified peroxiredoxin (Prdx) 1 as a CM728 target. Mechanistically, CM728 inhibited the peroxidase activity of Prdx1, induced oxidative stress, and promoted cell cycle arrest and apoptosis in TNBC cells.

## Methods and material

### CM728

CM728 (7,10-dihydroxy-12-(pyridin-3-yl)-12,13-dihydrobenzo[f]naphtho[2,3-b][1,4]oxazepine-6,11-dione) is a novel synthetic compound prepared by CEAMED S.A., Spain, with a purity of >98% (HPLC analysis) (it is disclosed in a European patent, application: EP21382995, compound number 24).

### Cell lines and cell culture

MDA-MB-231 (RRID: CVCL_0062), BT-549 (RRID: CVCL_1092), and Hs578T (RRID: CVCL_0332) TNBC cell lines; MRC-5 (RRID:CVCL_0440), a non-malignant lung fibroblast cell line; and HEK293T (RRID: CVCL_0063), an embryonic kidney cell line, were obtained from the American Type Culture Collection (ATCC). Cell line authentication was performed using short tandem repeats at the Hematology Department of the University Hospital of Salamanca. Cells were incubated at 37 °C in a humidified atmosphere with 5% CO_2_ and cultured for a maximum of 20 passages in Dulbecco's modified eagle medium (DMEM) (Biowest, Nuaille, France; L0106-500) (Hs578T, MDA-MB-231, MRC-5 and HEK 293T) or Roswell Park Memorial Institute 1640 (RPMI 1640) medium (Biowest, L0501-500) (BT-549) supplemented with 10% fetal bovine serum (FBS) (Gibco, Thermo Scientific, USA; 10270-106), 2 mM L-glutamine (Biowest, X0550-100), 100 mU/mL penicillin and 100 μg/mL streptomycin (Biowest, L0018-100). Anti-Mycoplasma treatment [ciprofloxacin (Kern Pharma, Barcelona, Spain; 38053390)] was regularly administered. Peripheral blood mononuclear cells (PBMC) were isolated from the blood of healthy donors. Twenty milliliters of heparin-anticoagulated blood were diluted 1:1 in saline solution. Diluted blood was added to tubes containing Ficoll-Paque PLUS (GE Healthcare Bio-Sciences AB, Uppsala, Sweden; 17-1440-02) and centrifuged at 400 × g for 40 min at 20 °C. The PBMC phase was transferred to a new tube and washed twice with a balanced salt solution. Finally, the cells were pelleted and resuspended in the RPMI 1640 medium. In all the experiments, cells treated with up to 0.1% dimethyl sulfoxide (DMSO) (Sigma-Aldrich, St Louis, MO, USA; D2650) corresponded to vehicle-treated (control) groups.

### MTT metabolism measurement

MDA-MB-231, BT-549, Hs578T, and MRC-5 cells were seeded into 96-well plates. The next day, the cells were treated as indicated for each case. PMBC cells were plated at a density of 200,000 cells/well and immediately incubated with phytohaemagglutinin (PHA) (Sigma-Aldrich, L8754) for 48 h. The cells were then treated with CM728 for 24 h. After treatments, 3-(4, 5-dimethylthiazolyl-2)-2, 5-diphenyltetrazolium bromide (MTT) (Sigma-Aldrich, M5655) was added at a final concentration of 0.3 µg/µL and incubated for 150 min at 37 °C. The medium was removed, cells were thoroughly mixed with 200 μL of DMSO (PanReac AppliChem, Barcelona, Spain; 191954.1611) and absorbance was measured at 570 nm using an iMark Microplate Reader (Bio-Rad, Hercules, CA, USA). Half maximal inhibitory concentration (IC_50_) values were calculated using the curve-fitting program GraphPad Prism 8 (GraphPad Software, CA, USA).

### Trypan blue exclusion assay

MDA-MB-231 cells were seeded at a density of 150,000 cells/well in 6-well plates. The next day, different concentrations of CM728 were added and the cells were further incubated for 24 or 48 h. For pulse exposure experiments, cells were treated with CM728 for the indicated time. The medium was then removed and replaced with fresh medium and the experiment was continued for 48 h. The cells were then trypsinized and collected at 500 × g at 4 °C for 10 min. Pellets were resuspended in cold phosphate-buffered saline (PBS) solution, followed by trypan blue staining, and counting of viable and non-viable cells using a TC10^TM^ Automated Cell Counter (Bio-Rad).

### Clonogenic assay

Colony formation was assessed using a previously described protocol (option A) 20.

### *In vitro* drug combination analysis

Cells were grown in 96-well plates and treated for 48 h (MDA-MB-231 and Hs578T) or 72 h (BT-549) with docetaxel (DTX) (Tocris, Ellisville, USA; 4056) or paclitaxel (PXT) (Sigma-Aldrich, T7402) alone or in combination with CM728. For drug combinations, a constant ratio of concentrations was used, in accordance with the IC_50_ values previously obtained for each drug. MTT metabolism data were analyzed using CalcuSyn software (version 2.0; Biosoft, Cambridge, UK) to obtain the combination index (CI). CI is an expression of drug pharmacological interactions and indicates synergy (CI < 0.9), additive effect (CI = 0.9-1.1) or antagonism (CI > 1.1).

### Toxicity evaluation

The acute toxicity of CM728 was evaluated using the Irwin test 21. Briefly, 8 female FVB mice (Charles River Code:207) were randomly distributed into two treatment groups: 1) Vehicle group (NMP/PEG400/0.1% methylcellulose in water for injection: 5/25/70), administered orally; and 2) CM728 in vehicle, administered orally (10 mg/kg). Animals were weighed and treated each day for 10 consecutive days. After each administration, clinical signs were gathered by two independent observers. After 10 days, the animals were euthanized by CO_2_ inhalation, and morphological changes in organs (liver, spleen, stomach, lungs) were recorded. The study was approved by the Bioethics committee of the University of La Laguna (CEIBA2018-0291). The experiment was performed according to the OECD guidelines for the testing of chemicals (N^o^ 407,452).

### Xenograft studies

Thirty-two female BALB/c nu/nu mice (seven weeks old) were purchased from Charles River Laboratories (Wilmington, MA, USA) (RRID: IMSR_JAX:000711). MDA-MB-231 cells (2 × 10^6^) were resuspended in 100 μL of DMEM containing 50% Matrigel and implanted into the mammary fat pads of the animals. When the tumors reached 60-70 mm^3^, the mice were randomly distributed into four groups: vehicle (control), CM728, DXT, or a combination of CM728 and DXT (CM728-DXT), and the treatment was initiated. CM728 (5-10 mg/kg) was administered orally 6 days/week and DXT (10 mg/kg) was injected into the intraperitoneal cavity 1 day/week. Tumor volume and body weight were measured twice per week. Tumor volume was calculated using the following formula: volume = (width^2^ × length)/2. After 28 days of treatment, mice were sacrificed by CO_2_ inhalation. The animal experimentation protocol was approved by the Bioethics Committee of the University of Salamanca, Spain (reference number 325).

### CM728 immobilization and identification of its targets

The immobilization of CM728 and identification of its targets were accomplished using a previously described method 22. Briefly, 5 mg of CM728 was dissolved in 50% dimethylformamide/50% 0.1 M Na_2_CO_3_ solution and incubated with 0.5 mg of freshly prepared epoxy-activated Sepharose 6B (GE Healthcare Life Sciences, Piscataway, NJ, USA; 17-0480-01), to obtain a CM728-coupled resin. A control resin was prepared using the same procedure but without CM728. Both resins were incubated with 27 mg of cell extracts from either MDA-MB-231 or BT-549 cells. The resulting complexes were retrieved by centrifugation, washed with lysis buffer, resuspended, boiled in loading buffer, and resolved by sodium dodecyl sulfate-polyacrylamide gel electrophoresis (SDS-PAGE). The gel was silver stained (Silver Stain Plus kit, Bio-Rad, 161-0449) and the bands of interest were excised, digested with trypsin, and analyzed using an Ultraflex MALDI‐TOF mass spectrometer (Bruker Daltonics, Bremen, Germany). Peptides were analyzed using the Mascot search engine (Matrix Science, London, UK) against the Swiss-Prot database, to identify the proteins. A protein score > 56 was considered significant (P < 0.05).

### ROS detection

MDA-MB-231 cells were seeded at a density of 250,000 cells/well in 6-well plates. After 24 h, the cells were treated with 1 µM CM728 for different times or preincubated with antioxidants (Table S1) for 2 h and then treated with CM728 for 30 min. Thirty minutes before stopping the treatment, 5 µM 2'-7'dichlorofluorescin diacetate (DCFH-DA) (Sigma-Aldrich, 35845) was added to the cells and incubated at 37 °C. For short treatments (less than 30 min), the cells were first incubated with DCFH-DA and then treated with CM728. The cells were trypsinized and collected at 500 × g for 5 min at 4 °C. Pellets were washed, centrifuged, and resuspended in cold PBS. DCF fluorescence was analyzed by flow cytometry using a BD FACSVerse cytometer (BD Biosciences, San Jose, CA, USA).

### Prdx1 knockdown

Prdx1 was downregulated as previously described 22. Five different short hairpin RNA (shRNA) sequences targeting Prdx1 were tested (sh#09, sh#10, sh#11, sh#12 or sh#13) (GE Dharmacon, Lafayette, CO, USA; RHS4533-EG5052). The three sequences that produced the highest knockdown levels were used in the experiments.

### Molecular Docking

Molecular docking studies were performed using Glide XP precision software 23-26. The methodology details are described in the Supplementary material.

### Western blot analyses

Cells were plated at a density of 500,000 - 750,000 cells in 100 mm dishes and treated as specified 24 h later. The cells were then washed with cold PBS-vanadate (1 mM) and lysed with cold radioimmunoprecipitation assay (RIPA) buffer (Thermo Scientific, 89900) containing protease and phosphatase inhibitors (Thermo Scientific, 78440). Lysates were centrifuged at 20,000 × g at 4 °C for 10 min, and the supernatants were collected. The protein concentration of the lysed extracts was measured using bicinchoninic acid (BCA) (Thermo Scientific, 23227). Electrophoresis sample buffer (Bio-Rad, 1610747) containing 10% 2-mercaptoethanol (Sigma-Aldrich, M6250) was added to 60 μg of extract. For non-reducing electrophoresis, the cells were incubated with cold PBS containing 25 mM N-ethylmaleimide (Sigma-Aldrich, E3876) for 30 min before lysis, and 2-mercaptoethanol was omitted from the electrophoresis sample buffer. Samples were separated by SDS-PAGE and transferred onto nitrocellulose (Bio-Rad, 170-4158) or PVDF membranes (Bio-Rad, 170-4156). After blocking non-specific binding with 1% blotto (Bio-Rad, 170-6404) and 1% bovine serum albumin (BSA) (PanReac AppliChem, A6588,0100), the membranes were incubated overnight with primary antibodies at 4 °C. Horseradish peroxidase (HRP)-conjugated secondary antibodies were used to detect the proteins. Protein bands were visualized with Clarity Western ECL substrate (Bio-Rad, 170-5061) using the ChemiDoc XRS System and the image analysis program Quantity One (Bio-Rad). Densitometric analyses were performed using the Image Processing Quantity One or ImageJ software (National Institutes of Health). Information regarding the antibodies used is provided in Table S2.

### Cell cycle and apoptosis assays

MDA-MB-231 cells were grown at a density of 300,000 cells/100 mm dish for 24 h. For cell cycle analysis, cells were treated with CM728 at either 0.5 or 1 µM, for 24 and 48 h periods. Then, the cells were treated with trypsin and collected at 500 × g at 4 °C for 10 min. The cell pellets were resuspended in cold PBS. To detect changes in the cell cycle phases and subG_0_ populations, cells were fixed with 70% cold ethanol overnight. The next day, the cells were centrifuged at 500 × g for 10 min at 4 °C, washed with PBS, and incubated with 100 μg/ml propidium iodide (Sigma-Aldrich, P4170) in the presence of RNAse (Sigma-Aldrich, R6513) in the dark for 1 h. For the detection of apoptosis, we used the Annexin V-FITC Apoptosis Detection Kit (BD Biosciences, 556547) according to the manufacturer's instructions. The samples were analyzed using a BD FACSVerse cytometer (BD Biosciences) and Flowing Software (version 2.5; Turku Bioscience, Turku, Finland).

### Caspase activity

Caspase activity was measured as previously described 27.

### Statistical analyses

Statistical analyses were performed using the GraphPad Prism 8 program (GraphPad Software). The results are presented as the mean ± SD of at least two independent experiments. The *in vivo* experiment was performed once, and the results are presented as the mean ± SEM. P-values were calculated using Student t test (two groups) or one-way or two-way ANOVA (more than two groups), followed by Dunnett's or Tukey's post-hoc tests. Statistical differences were considered significant when the p-value was less than 0.05.

## Results

### Effect of CM728 on TNBC cell viability

**Figure 1:**
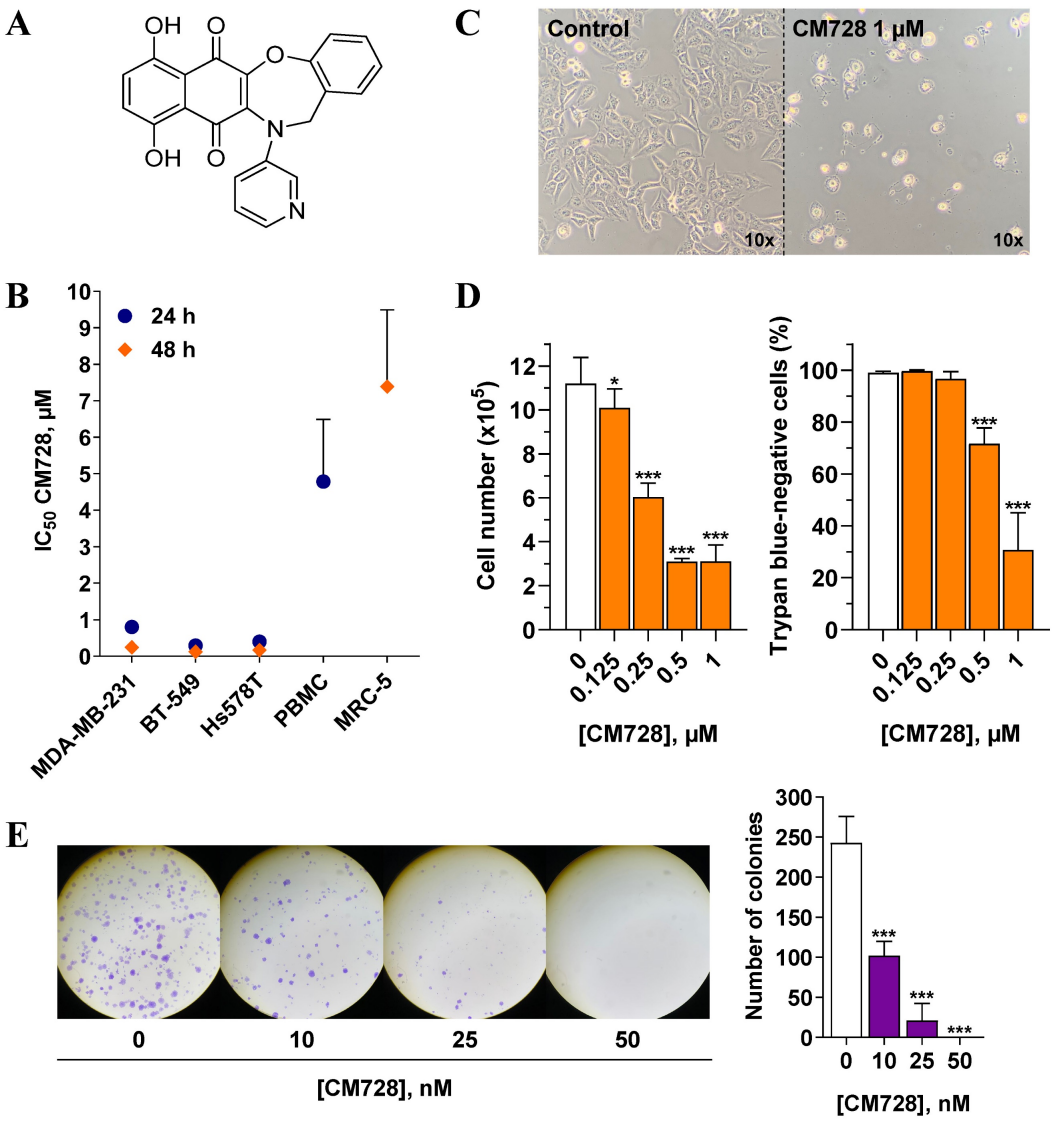
** Antitumor effects of CM728 in TNBC cell lines. A,** Chemical structure of CM728. **B,** Dose-dependent effect of CM728 on the viability of a set of cell lines after 24 h and 48 h of treatment assessed by MTT assay. IC_50_ values of at least two independent experiments are shown. **C,** General aspect of MDA-MB-231 cells after 24 h of incubation with 1 µM CM728, visualized using a phase-contrast microscope at 10x magnification. **D,** Effect of CM728 on cell number (left) and the percentage of trypan blue-negative cells (right) in MDA-MB-231 at 48 h (n=2). Cell counting was performed using trypan blue staining. **E,** Representative images (left), and counting (right) of colony formation after 9 days of treatment with CM728 in MDA-MB-231 cells (n=2). Statistical analyses were performed using one-way ANOVA and Dunnett's multiple comparison test. ^*^, *P* < 0.05 *versus* control; ^***^, *P* < 0.0001 *versus* control.

The molecular structure of CM728 is shown in Figure 1A. MTT assays were performed to estimate the effect of CM728 on TNBC cell viability. CM728 decreased the MTT metabolism at submicromolar IC_50_ concentrations in MDA-MB-231, BT-549 an d Hs578T cell lines (IC_50_= 0.24 ± 0.05 µM, 0.13 ± 0.02 µM, and 0.18 ± 0.06 µM at 48 h, respectively) (Figure 1B) This effect was significantly less potent in both PHA-stimulated PBMC (IC_50_ at 24 h = 4.79 ± 1.71 µM) and in the MRC-5 cells (IC_50_ at 48 h= 5.99 ± 0.77 µM), indicating a good degree of selectivity for TNBC cells over non-cancerous cells. The effect of napabucasin and taselisib was assessed in MDA-MB-231 (Figure S1A) and BT-549 cells (Figure S1B) as positive control. In both cell lines, these drugs decreased MTT metabolism in a concentration-dependent manner. Incubation of MDA-MB-231 cells with 1 µM CM728 for 24 h resulted in morphological changes (rounding), cell detachment, and a marked reduction in cell number (Figure 1C). Viable and non-viable MDA-MB-231 cells were counted using trypan blue staining. Concentrations of CM728 ≥ 0.125 µM decreased the number of cells (Figure 1D, left), while concentrations ≥ 0.5 µM caused a significant decrease in cell viability at 48 h (Figure 1D, right). In addition, clonogenic assays showed that MDA-MB-231 colonies were reduced by 58% after 9 days of treatment with concentrations as low as 10 nM of CM728 (Figure 1E). Napabucasin and taselisib also decreased the number of colonies at the concentrations used (Figure S1C). Notably, in this assay, CM728 was more potent than in the MTT experiments. Interestingly, pulse exposure experiments showed that a transient (30 min) exposure of MDA-MB-231 cells to CM728 (1 µM), followed by further incubation for 48h, caused a similar response to that observed after 48 h of sustained treatment (Figure S2A-B). This suggests that effective quantities of CM728 enter the cell rapidly, and trigger events that persist long time after a short exposure to the agent.

### CM728 synergizes with standard-of-care drugs

**Figure 2:**
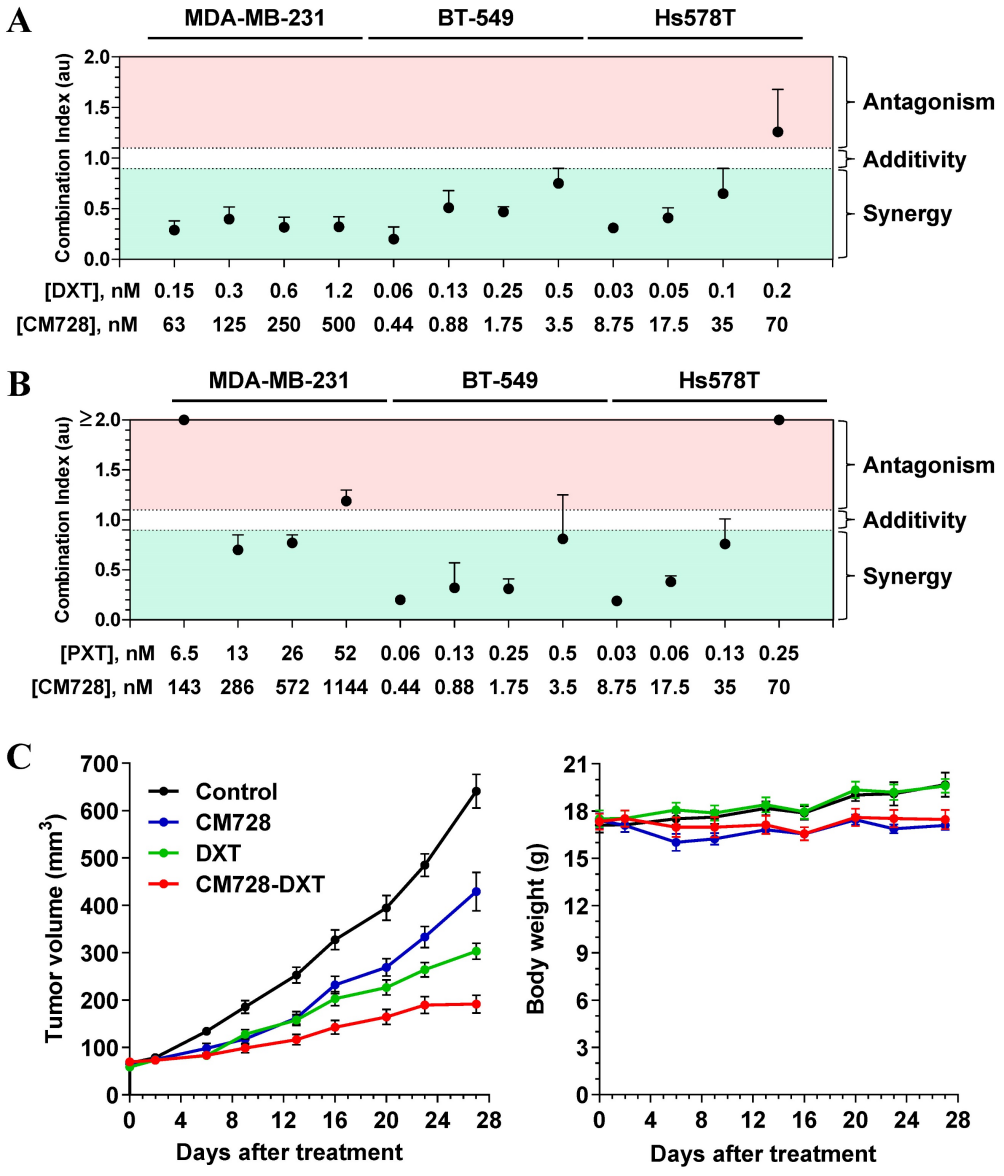
** Effect of the combination of CM728 and standard of care drugs on *in vitro* and *in vivo* models of TNBC. A-B,** Cells were treated with CM728 in combination with DXT (A) or PXT (B) for 48 h (MDA-MB-231 and Hs578T) or 72 h (BT-549). MTT metabolism assays were performed and analyzed using Chou-Talalay algorithm to obtain the combination index (n=2). **C,** Analysis of the growth of MDA-MB-231 xenograft tumors in response to vehicle, CM728, DXT, or the combination of CM728 and DXT (left), and monitoring of mice body weight (right) (n= 8 mice per group). CM728 (5-10 mg/kg) was administered orally 6 days/week and DXT (10 mg/kg) was injected into the intraperitoneal cavity 1 day/week. Statistical analyses were conducted using one-way ANOVA, and Tukey's multiple comparison test. Comparisons *versus* control (endpoint): CM728, *P* = 0.0002; DXT, *P* < 0.0001; CM728-DXT, *P* < 0.0001. Comparisons *versus* CM728 (endpoint): DXT, *P* = 0.0119; CM728-DXT, *P* < 0.0001. Comparisons *versus* DXT (endpoint): CM728-DXT, *P* = 0.0306.

Drug combination therapy is a common strategy for the treatment of several oncogenic disorders, including TNBC 28. Bearing that in mind, the possible potentiation of the effect of standard-of-care drugs by CM728 was explored. CM728 and DXT or PXT synergistically decreased the MTT metabolism in the three TNBC cell lines studied (Figure 2A and 2B).

Preliminary *in vivo* studies showed that CM728 was well tolerated. In particular, there was no loss of body weight in the CM728-treated group (Figure S3A-B).

To explore the *in vivo* antitumor efficacy of CM728 alone and in combination with taxanes, nude mice bearing orthotopically induced MDA-MB-231 tumors were treated with CM728, DXT, or in combination (CM728-DXT) for 27 days. At this later date point, both CM728 and DXT alone significantly decreased the rate of tumor growth (33.1%, P = 0.0002 and 52.7%, P < 0.0001, respectively, compared to the control). The combination of CM728-DXT showed an even stronger reduction of tumor volume (70.1%, P < 0.0001, compared to control, and 55.3%, P < 0.0001 and 36.8%, P = 0.0306, compared to CM728 and DXT alone, respectively) (Figure 2C, left). The body weight of animals treated with CM728 remained constant during the experiment (Figure 2C, right).

### Identification of CM728-bound proteins

**Figure 3:**
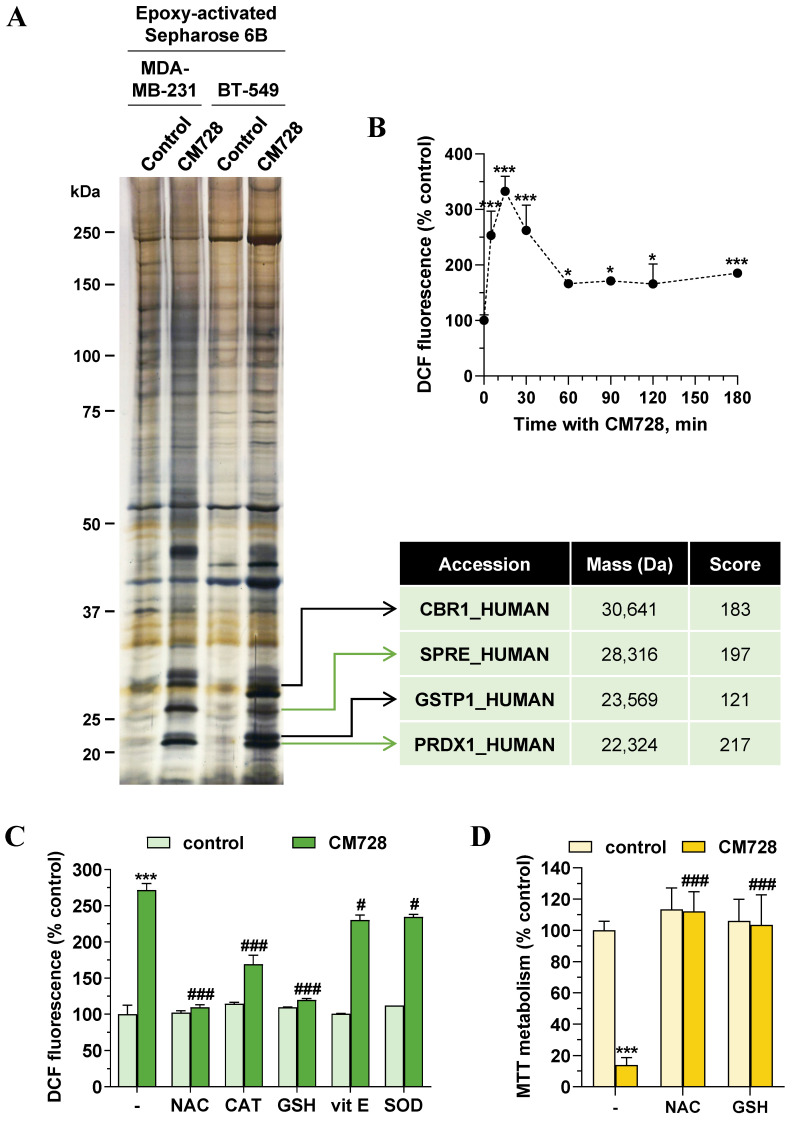
** Identification of potential CM728 targets. Relevance of ROS. A,** Identification of CM728-binding proteins in MDA-MB-231 or BT-549 cells. Cell lysates were incubated with uncoupled or CM728-coupled epoxy-activated Sepharose 6B and the resulting complexes were analyzed by SDS-PAGE and silver staining (left). Bands of interest were excised from the gel and analyzed using MALDI-TOF to the identification of proteins (right). **B,** Time-dependent induction of ROS by CM728 (1 µM) in MDA-MB-231 cells (n=2). **C,** Effect of 2 mM NAC, 1000 U/ml CAT, 1 mM GSH, 20 µM vit E, and 200 U/ml SOD in the presence or absence of CM728 (1 µM) on the percentage of DCFH-DA fluorescence in MDA-MB-231, at 30 min of treatment (n=2). **D,** Effect of 2 mM NAC and 1 mM GSH in the presence or absence of CM728 on cell viability, assessed by MTT metabolism at 48 h in MDA-MB-231 cells (n=2). Statistical analyses were conducted using one-way ANOVA, and Dunnett's **(B)** or Tukey's **(C-D)** multiple comparison tests. ^*^, *P* < 0.05 *versus* control; ^**^, *P* < 0.001 *versus* control; ^***^, *P* < 0.0001 *versus* control; ^#^, *P* < 0.05 *versus* CM728-treated cells; ^###^, *P* < 0.0001 *versus* CM728-treated cells.

To investigate the antitumor mechanism of action of CM728, we used a chemical proteomic approach to identify CM728 targets. To this end, CM728 was covalently coupled to an epoxy-activated Sepharose 6B resin, which was then incubated with MDA-MB-231, or BT-549 cell lysates. The resin coupled-CM728 interacted with several proteins related to ROS regulation, including: sepiapterin reductase (SPRE), glutathione S-transferase P (GSTP1), carbonyl reductase [NADPH] 1 (CBR1), and Prdx1, which showed the highest binding score (Figure 3A). Due to the role of these proteins in ROS metabolism, the effect of CM728 on ROS levels was evaluated using DCFH-DA. CM728 induced a rapid increase in ROS levels, which remained high for several hours in MDA-MB-231 cells (Figure 3B). This effect was completely abrogated by pretreatment with N-acetyl-L-cysteine (NAC) or glutathione (GSH), partially by catalase (CAT), and, to a lesser extent, by vitamin E (vit E) and superoxide dismutase (SOD) (Figure 3C). Additionally, pretreatment of MDA-MB-231 cells with NAC or GSH was shown to prevent CM728 from reducing their viability (Figure 3D), indicating that increased ROS levels were an important factor in the effects of CM728.

### Prdx1 in the mechanism of action of CM728

**Figure 4:**
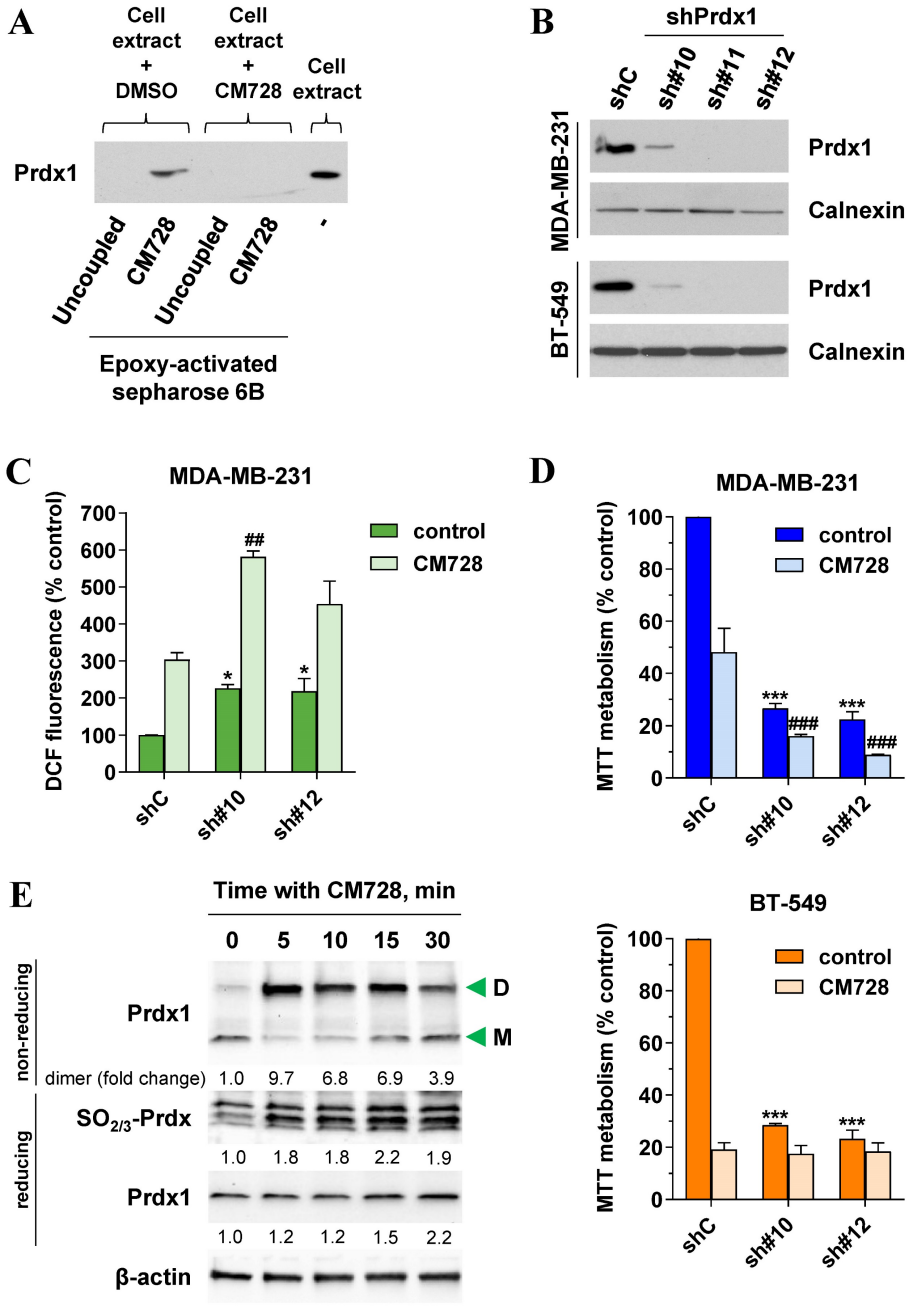
** Validation of Prdx1 as a target of CM728 in TNBC cells. A,** Identification of Prdx1 as a target of CM728 in MDA-MB-231 cells by western blotting. Cell extracts were used as a positive control. **B,** Cell extracts of Prdx1 knockdown MDA-MB-231 and BT-549 cells were used to evaluate the expression levels of Prdx1 by immunoblotting. Calnexin was used as a loading control (shC: shRNA transfection control; sh#10, sh#11, and sh#12: shRNA sequences targeting Prdx1). **C,** Effect of Prdx1 downregulation on the percentage of DCF fluorescence in MDA-MB-231 cells untreated or treated with 1 µM CM728 for 30 min. **D,** Effect of Prdx1 knockdown on MTT metabolism of MDA-MB-231 and BT-549 cells, untreated or treated with 0.2 or 0.15 µM CM728, respectively, at 48 h. **E,** Western Blot analysis of the monomer (M) and dimer (D) forms of Prdx1, or the levels of SO_2/3_-Prdx in response to CM728 (1 µM) in MDA-MB-231 cells. β-actin was used as a loading control. Densitometry quantification from immunosignal values is shown below the bands. Values are relativized to the loading control. Representative images of two independent experiments are shown. Statistical analyses were conducted using one-way ANOVA followed by Dunnett's multiple comparison test. ^*^, *P* < 0.05 *versus* vehicle-treated shC cells; ^***^, *P* < 0.0001 *versus* vehicle-treated shC cells; ^##^, *P* < 0.001 *versus* CM728-treated shC cells; ^###^, *P* < 0.0001 *versus* CM728-treated shC cells.

The binding of CM728 to Prdx1 was further validated using western blotting (Figure 4A). As expected, when MDA-MB-231 lysates were pretreated with free CM728, the excess free drug prevented the interaction between Prdx1 and the resin-bound CM728 (Figure 4A and Figure S4A) but had a little effect on the other protein bands (Figure S4A).

**Figure 5:**
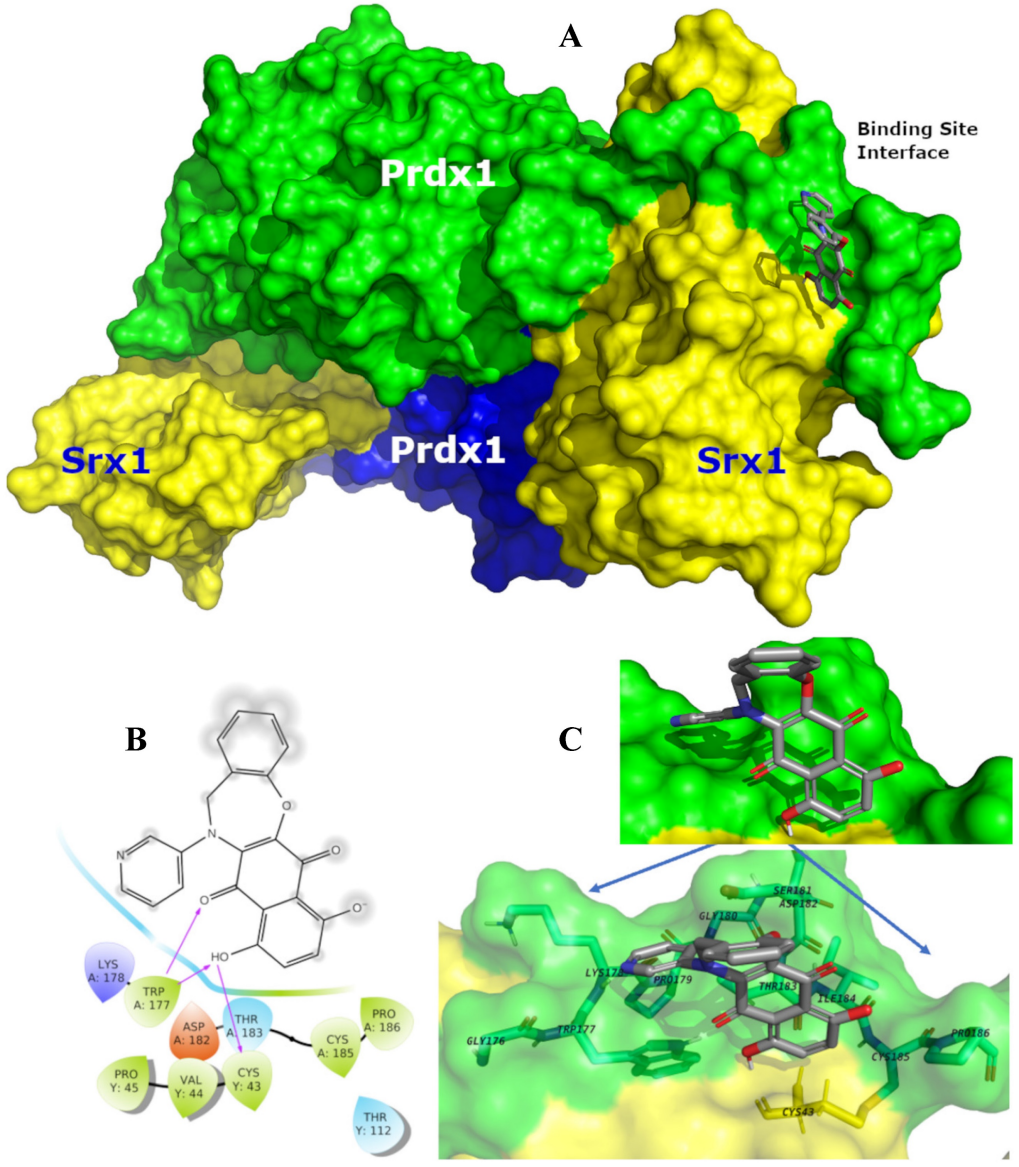
**Predicted binding mode of CM728 with Prdx1**. **A,** Surface representation of the sulfiredoxin 1 (Srx1)-Prdx1 complex, illustrating the binding site interface and backside interfaces (*blue* surface). In this view the CM728 is interacting with the Prdx1 (*green-ligth* surface) and less with Srx1 (*yellow* surface). **B,** Individual interactions between CM728 and amino acid residues 4 Å around the ligand, highlighting the interactions with the residues Cys43 and TRP177. **C,** Binding mode of CM728 (*gray* sticks) into Prdx1 binding site interface (PDB code: 3HY2) represented as *green-light* surface. Only the main residues are displayed (sticks) and labeled.

Prdx1 was downregulated using shRNA to explore its relevance in TNBC cells. All three shRNA sequences (sh#10, sh#11, and sh#12) effectively silenced Prdx1 in MDA-MB-231 and BT-549 cells (Figure 4B). Knockdown of Prdx1 in MDA-MB-231 cells significantly increased ROS levels compared to the shRNA transfection control (Figure 4C), while the addition of CM728 to Prdx1 knockdown cells caused an additional increase in ROS levels (Figure 4C). Knockdown of Prdx1 decreased the ability of cells to metabolize MTT in both cell lines and also sensitized MDA-MB-231 cells to the effect of CM728 (Figure 4D). CM728 led to a dramatic and rapid increase in the amount of the dimeric (disulphide-bridged) form of Prdx1, which is an inactive catalytic state of the protein (Figure 4E, Figure S4B). In addition, CM728 increased the amount of hyperoxidized peroxiredoxin (SO_2/3_-Prdx), in which the catalytic site is also inactive (Figure 4E and Figure S4B). Together these results indicate that CM728 can rapidly convert catalytically active Prdx1 into inactive forms. Molecular docking studies were performed to assess the potential binding mode of CM728 to Prdx1. Predicted binding of CM728 to Prdx1 lowered the energy of the system by 3 kcal/mol, indicating a considerable affinity for Prdx1 (Figure 5A-C). Hydrogen bonds were predicted to be formed between the 1,4-NQ core and Cys43 and Trp177 of Prdx1, while other favorable hydrophobic interactions between CM728 and Prdx1 were also predicted.

### CM728 modulates various signaling pathways

**Figure 6:**
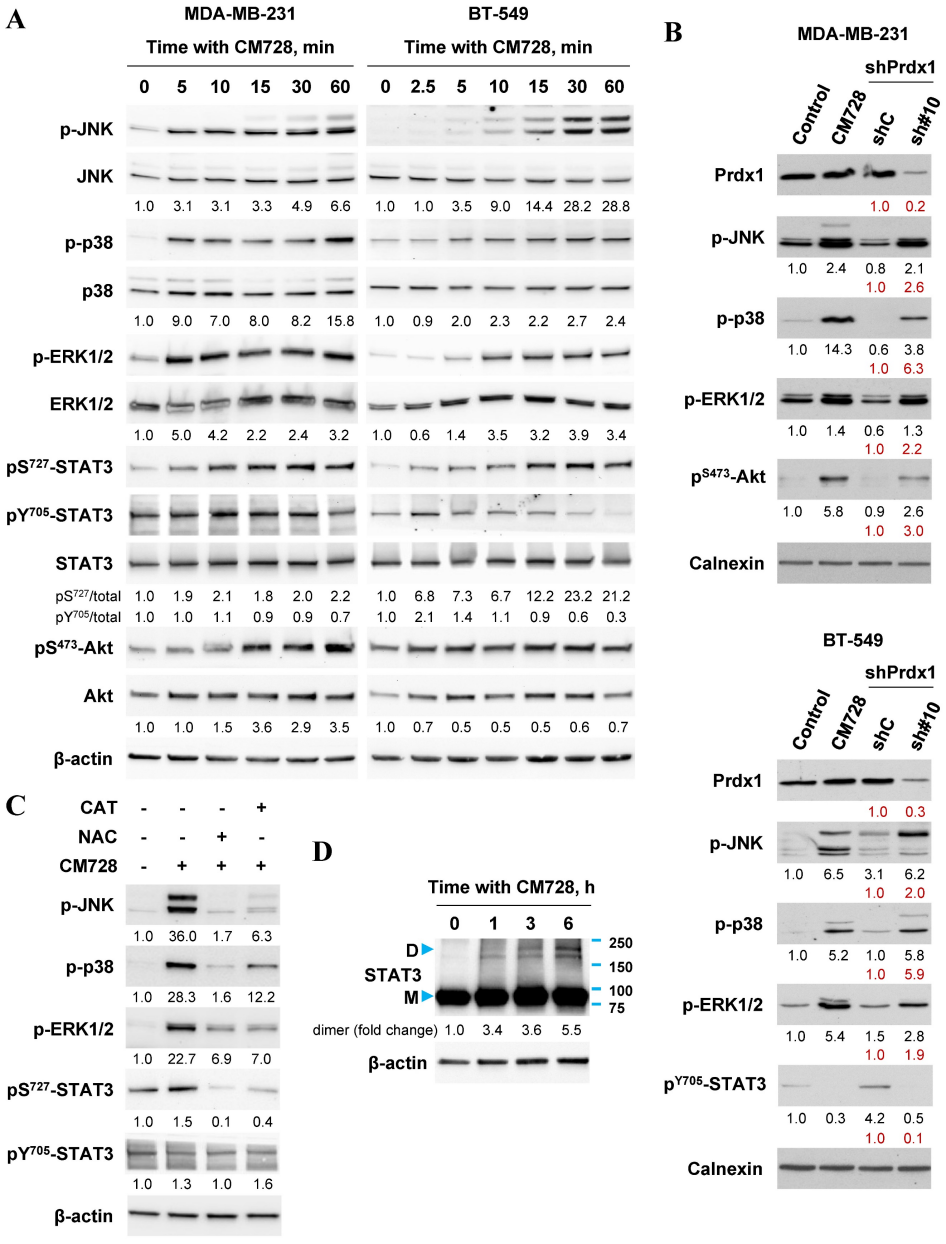
**Effect of CM728, or Prdx1 knockdown on MAPKs, STAT3 and Akt in TNBC cell lines. A,** Time-dependent effect of CM728 (1 µM) on MAPKs, STAT3 and Akt phosphorylation in MDA-MB-231 (left) and BT-549 cells (right). **B,** Immunoblot of phosphorylated MAPKs, STAT3 and Akt in Prdx1-knocked down TNBC cells. Lysates from cells treated with CM728 (5 µM) for 3 h were used as a positive control. **C,** Analysis of MAPK and STAT3 phosphorylation in MDA-MB-231 cells that were preincubated with 2 mM NAC or 1000 U/ml catalase for 2 h and then treated with 1 µM CM728 for 12 h. **D,** Dimerization of STAT3 in response to CM728 (1 µM) (D: dimer; M: monomer). β-actin or calnexin was used as loading controls. Densitometry quantification from immunosignal values is shown below the bands. Phosphorylated forms of proteins are relativized to the total amount of the corresponding proteins. In all the cases values are relativized to the loading control (A, C). In red, band densitometry compared to shC. Representative images of two independent experiments are shown.

A wide variety of signaling pathways can be modulated once oxidative stress has been induced. Common pathways include, the mitogen-activated protein kinase (MAPK) cascade 15, 17, 29, the STAT3 pathway 30 and the phosphatidylinositol 3-kinase (PI3K)/protein kinase B (Akt) 17, 31 pathways amongst others. The effects of CM728 on these pathways were investigated in two TNBC cell lines. CM728 induced a rapid and sustained increase in the phosphorylation of c-Jun N-terminal kinase (JNK) and p38 MAPK proteins in MDA-MB-231 and BT-549 cells (Figure 6A and Figure S5). In addition, CM728 induced phosphorylation of extracellular signal-regulated kinase (ERK1/2) in both cell lines, (Figure 6A and Figure S5). The phosphorylation of JNK, p38 MAPK, and ERK1/2 was inhibited by either NAC or CAT (Figure 6C), again highlighting the importance of oxidative stress to their activation. Knockdown of Prdx1 also induced phosphorylation of JNK, p38 MAPK, and ERK1/2 in both cell lines (Figure 6B). Interestingly, CM728 induced a rapid phosphorylation of STAT3 at Ser^727^, followed by a later dephosphorylation of STAT3 at Tyr^705^, in both cell lines (Figure 6A and Figure S5). The increase in pS^727^-STAT3 level induced by CM728 was prevented by NAC and CAT (Figure 6C). CM728 also induced the formation of covalently linked STAT3 dimers in MDA-MB-231 cells (Figure 6D), a transcriptionally inactivated state of the protein 30, 32. Altogether, the above data indicate that CM728 inactivates STAT3 in a ROS-dependent manner. Interestingly, Prdx1 downregulation decreased the levels of pY^705^-STAT3 in BT-549 cells (Figure 6B). Finally, regarding the PI3K/Akt cascade, both CM728 treatment (Figure 6A and Figure S5) and Prdx1 knockdown (Figure 6B) increased pS^473^-Akt levels in MDA-MB-231 cells. However, in BT-549, CM728 decreased Akt phosphorylation at Ser^473^ (Figure S5). Overall, the data suggests that inactivation, or downregulation, of Prdx1 leads to the rapid activation of several signaling pathways *via* oxidative stress.

### CM728 modulates the cell cycle, and cell death machinery

**Figure 7:**
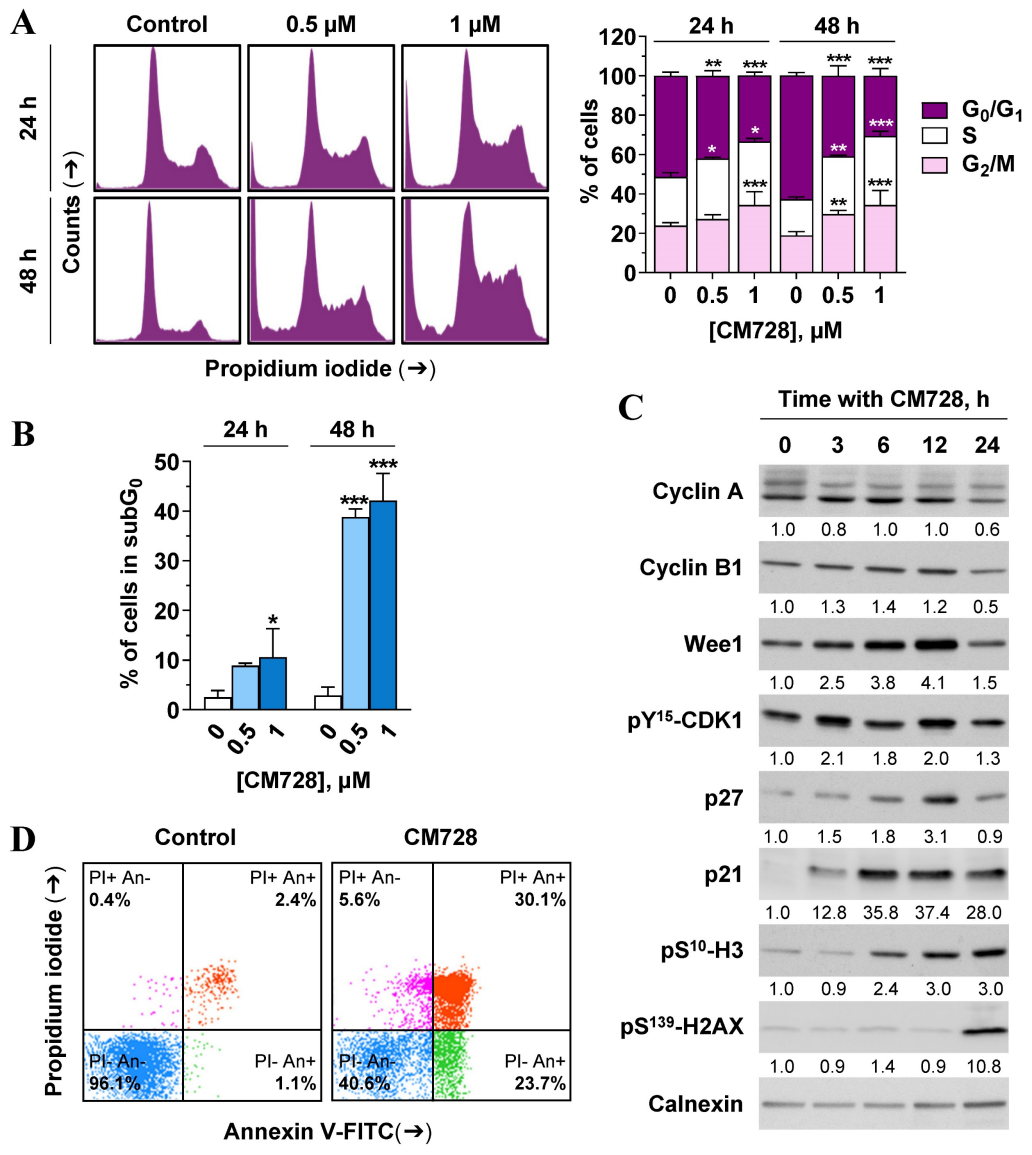
** Cell cycle distribution and apoptosis in response to CM728 in MDA-MB-231 cells. A-B**, Cell cycle was analyzed by propidium iodide staining and flow cytometry at 24 and 48 h of treatment with 0.5 and 1 µM CM728. **A,** Representative histogram (left), and percentage of cells in each cycle phase (right); and,** B,** percentage of subG_0_ population are shown (n=2). **C,** Immunoblot study of some relevant proteins involved in the cell cycle progression after treatment with 1 µM CM728 for the indicated times. Calnexin was used as a loading control. Densitometry quantification from immunosignal values is shown below the bands. Values are relativized to the loading control. **D,** Annexin V/propidium iodide double staining and flow cytometry analyses in response to 48 h of incubation with CM728 (1 µM). Representative images of three independent experiments are shown. PI: propidium iodide; An: annexin V. Statistical analyses were performed using two-way ANOVA followed by Tukey's post-hoc test (A) or using one-way ANOVA, and Dunnett's multiple comparison test (B). ^*^, *P* < 0.05 *versus* control; ^**^, *P* < 0.001 *versus* control; ^***^, *P* < 0.0001 *versus* control.

Since CM728 caused a marked reduction in cell viability, we wished to assess its effects on the cell cycle and cell death machinery. CM728 caused an accumulation of MDA-MB-231 cells in their S and G_2_/M phases (Figure 7A). Consistent with these data, CM728 increased Wee1-like protein kinase (Wee1) and pY^15^-cyclin-dependent kinase 1 (CDK1) levels, induced the phosphorylation of mitotic histone H3 (H3) at Ser^10^, augmented the levels of p27 and caused a sustained increase in p21 WAF1/Cip1 (p21) (Figure 7C). Interestingly, at later incubation times, CM728 produced a significant increase in γ-H2A histone family member (H2AX) levels (Figure 7C), indicative of DNA double-strand breaks 33.

**Figure 8:**
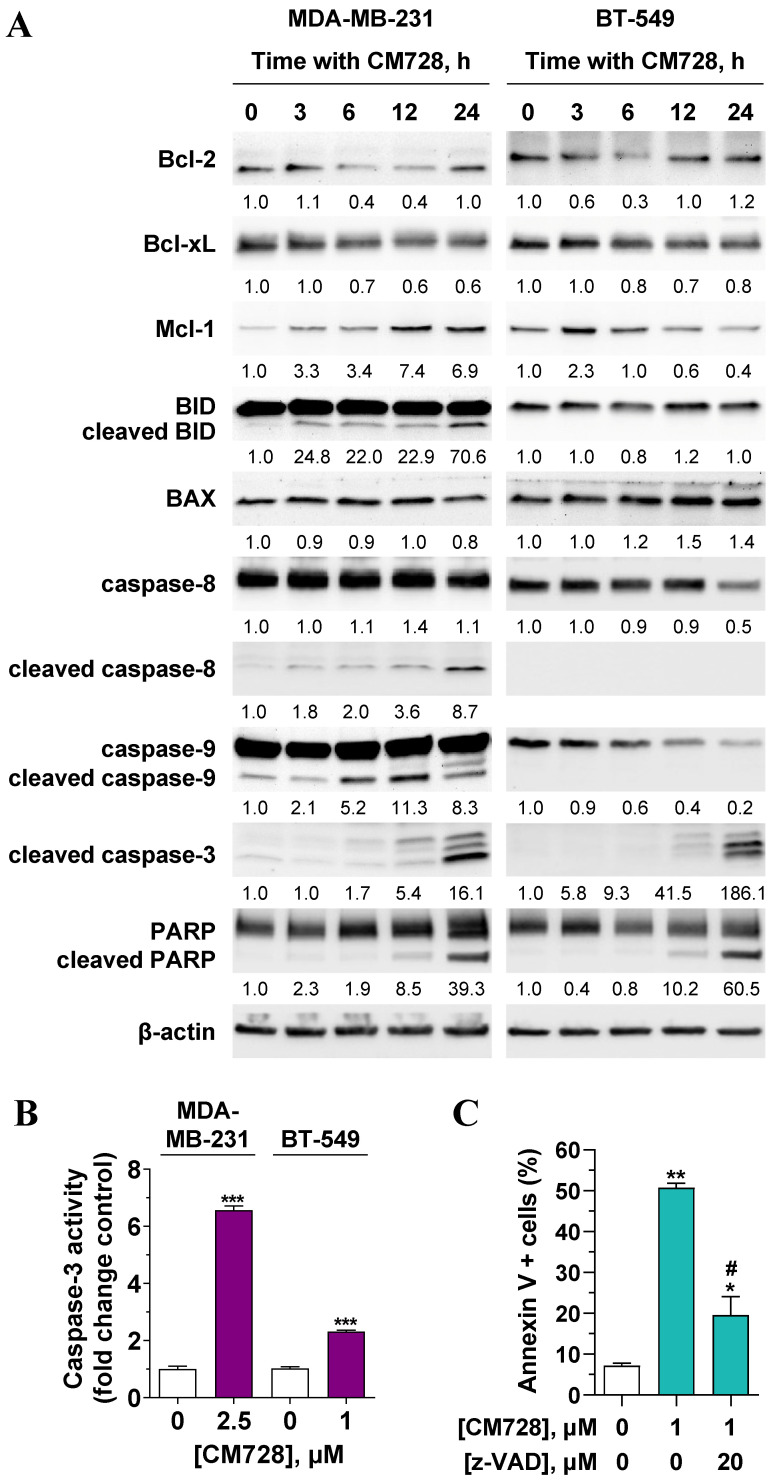
Effect of CM728 on the extrinsic and intrinsic apoptotic pathways in TNBC cells.** A,** Time-course analyses of the effect of 1 µM CM728 on apoptosis-related proteins in MDA-MB-231 and BT-549 cells by Western blot. β-actin was used as a loading control. Densitometry quantification from immunosignal values is shown below the bands. Values are relativized to the loading control. Representative images of two independent experiments are shown. **B,** Caspase-3 activity in response to CM728 in MDA-MB-231 and BT-549 cells at 24 h of treatment. **C,** Effect of z-VAD-fmk on the percentage of annexin V-positive population in MDA-MB-231, analyzed by flow cytometry. Cells were preincubated with z-VAD-fmk for 1 h and then treated with CM728 during 48 h (n=2). Statistical analyses were conducted using the Student t test (B) or one-way ANOVA followed by Tukey's post-hoc test (C). ^*^, *P* < 0.05 *versus* control; ^**^, *P* < 0.001 *versus* control;^ ***,^ *P* < 0.0001 *versus* control; ^#^, *P* < 0.05 *versus* CM728-treated cells.

CM728 strongly increased the percentage of subG_0_ cells (Figure 7A-B), and the number of cells in the early and late phases of apoptosis (Figure 7D). Notably, pretreatment of MDA-MB-231 cells with the antioxidant NAC before CM728 treatment, prevented the cell cycle arrest, substantially reduced the number of cells in subG_0_, reduced the activation of both p21 and pS^139^-H2AX, and generated fewer apoptotic cells (Figure S6A-D). CM728 induced the proteolytic cleavage of both caspase-8 and -9 in MDA-MB-231 cells (Figure 8A), while in BT-549 cells CM728 caused a decrease in procaspase 8 and procaspase 9 levels particularly after 24 h (Figure 8A). CM728 also induced cleavage of procaspase-3 and its substrate PARP (Figure 8A), the latter effect being significantly reduced by pretreatment with NAC (Figure S6C). Additionally, CM728 also increased caspase-3 activity (Figure 8B). CM728 diminished the levels of anti-apoptotic proteins B-cell lymphoma 2 (Bcl-2) and B-cell lymphoma-extra large (Bcl-xL) in both the cell lines. However, the levels of anti-apoptotic myeloid cell leukemia-1 (Mcl-1) were increased in MDA-MB-231 cells but decreased in BT-549 cells (Figure 8A). Interestingly, the Mcl-1 inhibitor S63845 had no effect on MDA-MB-231 cell viability by itself (Figure S7A), but it enhanced the decrease in viability caused by CM728 alone (Figure S7B). Regarding pro-apoptotic proteins, CM728 induced truncated and active forms of BH3 interacting-domain death agonist (BID) in MDA-MB-231 cells and increased Bcl-2-associated X protein (BAX) levels in BT-549 cells (Figure 8A). z-VAD-fmk, a pan-caspase inhibitor, considerably reduced the percentage of apoptotic cells induced by CM728 in MDA-MB-231 cells, corroborating the implication of caspases in the mechanism of cell death (Figure 8C).

## Discussion

The lack of relevant molecular targets for TNBC has hampered the development of effective treatments. Although a few target-specific drugs have been approved for clinical use, the identification of TNBC vulnerabilities and the emergence of new drugs are essential. In this scenario, a chemical library of more than one-hundred compounds based on a quinone-fused oxazepine scaffold was synthesized by CEAMED S.A. Among these, CM728 was identified as the leader compound. In the current study, we report the antitumor action of CM728 in TNBC both *in vitro* and *in vivo*.

In pursuit of CM728 potential targets, chemical proteomic approaches were carried out, and Prdx1 was found to be a target. That Prdx1 could be a direct target of CM728 was also indicated by the molecular docking studies. Prdxs are a family of six cysteine-dependent peroxidases whose main enzymatic activity is to catalyze the reduction of H_2_O_2_ to water 34. Given the homology among certain members of this family, it would be not surprising if CM728 could interact with several Prdx proteins. However, it is relevant to indicate that our chemical-proteomic studies were only able to identify Prdx1. Together with their relevant role as ROS regulators, other functions of Prdxs, such as chaperone activity, have been described 34, 35. In breast cancer, Prdx1 is overexpressed and contributes to cell survival; therefore, its inhibition may be a potential therapeutic strategy 36, 37. We showed that CM728 rapidly increased both covalently linked dimers of Prdx1 and hyperoxidized Prdx, provoking a negative effect on the capability of Prdx1 to deactivate excessive cellular levels of ROS. CM728-induced oxidative stress was shown to be responsible for the antitumor effect by the use of antioxidants.

We corroborated the importance of Prdx1 in TNBC redox homeostasis and survival as Prdx1 downregulation led to elevation of ROS levels and inhibition of cell viability. Based on this, the lack of Prdx1 activity could be considered a vulnerability of the MDA-MB-231 and BT-549 cells. Inhibition of Prdx1 has been reported to stimulate apoptosis in hepatocellular 38, lymphoma 39, and lung cell lines 40 by increasing ROS levels and/or modulating kinase signaling pathways, such as MAPKs, Abelson tyrosine-protein kinase 1 (c-ABL), Myc proto-oncogene protein (c-MYC), apoptosis signal-regulating kinase 1 (ASK1), or STATs. Ye et al. demonstrated that inhibition of Prdx1 by the natural product frenolicin induces cancer cell cytotoxicity in a variety of human-derived cell lines, including one TNBC line. Furthermore, they reported that a derivative of frenolicin reduced the growth of colorectal tumors *in vivo* 41. In addition, redox systems could be implicated in breast cancer resistance to DXT. In particular, elevated gene expression of Prdx1 has been found in breast cancer tumors of patients with no response to DXT 42. Our *in vitro* and *in vivo* results were consistent with these findings.

MAPKs are key signal transduction pathways implicated in numerous cellular processes, such as proliferation, survival, and apoptosis 43. The marked and sustained phosphorylation of JNK and p38 MAPK in response to CM728 or Prdx1 downregulation-induced oxidative stress probably mediates the activation of apoptosis 29. STAT3 has been suggested to be a potential target in TNBC 44. Although the connection between Prdx1 and STAT3 has not been reported, the inhibition of STAT3 activity by CM728 could be mediated by Prdx1 because this effect was mimicked by Prdx1 knockdown. The connection between loss of activity of the Prdx2/thioredoxin-1/thioredoxin reductase 1 system, oxidative stress, and STAT3 activity has been previously described 30, 32. Moreover, napabucasin exerts antitumor activity via oxidative stress and STAT3 inhibition 45, 46. Altogether, the similarity between the response profile observed after treatment with CM728 and the downregulation of Prdx1 suggests a central role for Prdx1 in the mechanism of action of CM728.

The growth inhibitory effect caused by CM728 was associated with cell cycle blockage at the S and G_2_/M phases, as supported by increases in Wee1 and Y^15^-CDK1 phosphorylation, both associated with the accumulation of cells in the S phase and the G_2_/M transition impairment 47; the phosphorylation of histone H3 at Ser^10^ is related to chromosomal segregation instability 48; and an increase in p21, a multi-cyclin-CDK inhibitor that participates in speed regulation throughout all phases of the cell cycle 49. Moreover, the increase in γ-H2AX levels in response to CM728 indicated a chromatin remodeling process provoked by chromosomal double-strand breaks, suggesting that the cells were unable to overcome the cell cycle checkpoint and died 33. In fact, the drug induced apoptosis, as indicated by an increase in both the subG_0_ population and the number of annexin V-positive cells. Moreover, this process was caspase-dependent, as z-VAD-fmk impeded the apoptotic action of CM728. The increase in Mcl-1 levels after CM728 treatment in MDA-MB-231 cells could be interpreted as a resistance mechanism, as the specific Mcl-1 inhibitor S63845 potentiated the effect of CM728 on cell viability 50, 51. In conclusion, we report that the novel benzoxazepine-naphthoquinone chimeric compound CM728 exerts potent antitumor effects against TNBC. Using a chemical-proteomic approach, we identified Prdx1 as a cellular target of this drug. Mechanistically, targeting peroxiredoxin-1 with CM728 or specific shRNAs resulted in prolonged increases in ROS levels and, consequently, the modulation of JNK, p38 MAPK and STAT3 activity. CM728 causes DNA damage, cell cycle arrest, and apoptosis. Finally, we show that CM728 potentiates the antitumor action of standard-of-care drugs used in the breast cancer clinics. Taken together, our results offer the possibility of further exploring the validity of CM728 or other products that target Prdx1, alone or in combination with current chemotherapeutics, as a potential novel strategy to treat TNBC.

## Supplementary Material

Supplementary methods, figures and tables.

## Abbreviations

Akt: protein kinase B
ASK1: apoptosis signal-regulating kinase 1
BAX: Bcl-2-associated X protein
BCA: bicinchoninic acid
Bcl-xL: B-cell lymphoma-extra large
Bcl-2: B-cell lymphoma 2
BID: BH3 interacting-domain death agonist
BRCA: breast cancer genes
BSA: bovine serum albumin
c-ABL: Abelson tyrosine-protein kinase 1
c-MYC: Myc proto-oncogene protein
CAT: catalase
CBR1: carbonyl reductase [NADPH] 1
CDK1: cyclin-dependent kinase 1
CDK8: cyclin dependent kinase 8
CI: combination index
DCFH-DA: 2'-7'dichlorofluorescin diacetate
DMEM: Dulbecco's modified eagle medium
DMSO: dimethyl sulfoxide
DTX: docetaxel
ER: estrogen receptor
ERK1/2: extracellular signal-regulated kinase
FBS: fetal bovine serum
GSH: glutathione
GSTP1: glutathione S-transferase P
HER2: human epidermal growth factor receptor 2
HRP: horseradish peroxidase
H2AX: H2A histone family member
H3: histone H3
IC50: half maximal inhibitory concentration
JAK: Janus kinase
JNK: c-Jun N-terminal kinase
MAPK: mitogen-activated protein kinase
Mcl-1: myeloid cell leukemia-1
MTT: 3-(4, 5-dimethylthiazolyl-2)-2, 5-diphenyltetrazolium bromide
NAC: N-acetyl-L-cysteine
PARP: poly (ADP-ribose) polymerase
PBMC: peripheral blood mononuclear cells
PBS: phosphate-buffered saline
PD-L1: programmed cell death ligand 1
PD-1: programmed cell death protein 1
PHA: phytohaemagglutinin
PI3K: phosphoinositide 3-kinase
Prdx(s): peroxiredoxin(s)
PXT: paclitaxel
p21: p21 WAF1/Cip1
RIPA: radioimmunoprecipitation assay
ROS: reactive oxygen species
RPMI 1640: Roswell Park Memorial Institute 1640
SDS-PAGE: sodium dodecyl sulfate-polyacrylamide gel electrophoresis
SOD: superoxide dismutase
SPRE: sepiapterin reductase
STAT3: signal transducer and activator of transcription 3
TNBC: triple-negative breast cancer
Vit E: vitamin E
Wee1: Wee1-like protein kinase
1,4-NQ(s): 1,4-naphthoquinone(s)
