# Supplementary material for: Chemical-proteomics Identify Peroxiredoxin-1 as an Actionable Target in Triple-negative Breast Cancer
Source: Int J Biol Sci. 2023 Mar 13;19(6):1731–47. doi: 10.7150/ijbs.78554 (PMC10092761; doi:10.7150/ijbs.78554)
Supplement: Supplementary file 1 — Supplementary methods, figures and tables. [file ijbsv19p1731s1.pdf]

Supplementary Material

Chemical-proteomics Identify Peroxiredoxin-1 As An Actionable Target In Triple-negative Breast Cancer

Elena Spínola-Lasso [1], Juan Carlos Montero [2], Roberto Jiménez-Monzón [3], Francisco Estévez [1], José Quintana [1], Borja Guerra [4,5], Khaled M. Elokely [6], Francisco León [7], Henoc del Rosario [1], Leandro Fernández-Pérez [4,5], Manuel Rodríguez López [8], Bonifacio Nicolás Díaz-Chico [1,3,8], Grant McNaughton-Smith [8], Atanasio Pandiella [2*], Juan Carlos Díaz-Chico [1*]

Affiliations:

[1] Instituto Universitario de Investigaciones Biomédicas y Sanitarias (IUIBS), Departamento de Bioquímica y Biología Molecular, Fisiología, Genética e Inmunología, Universidad de Las Palmas de Gran Canaria, The Canary Islands, Spain.

[2] Institute of Biomedical Research of Salamanca (IBSAL), Instituto de Biología Molecular y Celular del Cáncer-CSIC and CIBERONC, Salamanca, Spain.

[3] Instituto Canario de Investigación del Cáncer (ICIC), The Canary Islands, Spain.

[4] Instituto Universitario de Investigaciones Biomédicas y Sanitarias (IUIBS), Farmacología Molecular y Traslacional, Departamento de Ciencias Clínicas, Universidad de Las Palmas de Gran Canaria, The Canary Islands, Spain.

[5] Unidad de Biomedicina asociada al CSIC, Instituto Universitario de Investigaciones Biomédicas y Sanitarias (IUIBS), Universidad de Las Palmas de Gran Canaria, The Canary Islands, Spain and Instituto de Investigaciones Biomédicas “Alberto Sols" CSIC - Universidad Autónoma de Madrid, Madrid, Spain

[6] Institute for Computational Molecular Science and Department of Chemistry, Temple University, Philadelphia, USA.

[7] Department of Drug Discovery and Biomedical Sciences, College of Pharmacy, University of South Carolina, Columbia, USA.

[8] Centro Atlántico del Medicamento S.A. (CEAMED S.A), La Laguna, The Canary Islands, Spain.

* Corresponding authors: Prof. Atanasio Pandiella, Tel./Fax: +34 923294815, E-mail: atanasio@usal.es; Prof. Juan Carlos Díaz-Chico, Tel.: +34 928451445, E-mail: juancarlos.diaz@ulpgc.es.

Supplementary Material:

- Supplementary Methods
- Supplementary Tables
- Supplementary Figures

Supplementary Methods

Molecular Docking

The crystal structure of the dimeric Prdx1 in complex with sulfiredoxin (PDB ID: 3HY2) [1] was downloaded from the RCSB database [2]. We choose to use this complex structure as the basis for our modeling studies because of its reasonable atomic resolution. We used Maestro's Protein Preparation Wizard [3, 4] to hone the structure. Prime [5 – 7] was used to add missing atoms and residues to the protein structure and complete the incomplete parts of the loops. After the original hydrogen atoms were deleted, the bond orders of the amino acid residues and ligands were readjusted. The degree of protonation was altered to achieve the desired pH value. Only water molecules within 5 Å from ligands and active sites were maintained to save calculation time. Hydrogen bonds were sampled at pH 7.4 using PROPKA [8], with the water molecule orientations in the active site altered to achieve this value. The OPLS3e force field [9] was used to geometrically refine the protein complex until an RMSD of 0.3 Å was reached via restricted reduction with heavy atom convergence, therefore resolving any remaining steric conflicts. The receptor grids were prepared using the OPLS3e force field. SiteMap [10 – 12] is utilized for locating druggable Prx1 areas. The sensitivity of the receptor was enhanced by boosting the Van der Waals radii of its non-polar atoms by a factor of 1. A requirement of 0.25 or less for the absolute partial atomic charge for a non-polar atom was considered. The centroids of the ligand binding regions have been used to establish the centers of 20 $Å^3$ grids. No constraints were imposed on rotations in any of the binding locations, and no receptor grids were used. Glide [13 – 16] was used to dock the fully prepared ligands into the receptor grids in Maestro with XP docking precision. Setting the scaling factor for Van der Waals radii of the ligand's non-polar atoms to 0.8 and the absolute partial atomic charge cut-off to 0.15 allowed for the exploration of a softer potential during docking simulations. All poses were minimized following docking.

References

1. Jönsson TJ, Johnson LC, Lowther WT. Protein engineering of the quaternary sulfiredoxin.peroxiredoxin enzyme.substrate complex reveals the molecular basis for cysteine sulfinic acid phosphorylation. J Biol Chem 2009; 284:33305-10. https://doi.org/10.1074/jbc.M109.036400
2. Rose PW, Prlić A, Bi C, et al. The RCSB Protein Data Bank: views of structural biology for basic and applied research and education. Nucleic Acids Res 2015; 43(Database issue):D345-56. https://doi.org/10.1093/nar/gku1214

3. Sastry GM, Adzhigirey M, Day T, et al. Protein and ligand preparation: parameters, protocols, and influence on virtual screening enrichments. J Comput Aided Mol Des 2013; 27:221-34. https://doi.org/10.1007/s10822-013-9644-8
4. Schrödinger Release 2021-3: Protein Preparation Wizard; Epik, Schrödinger, LLC, New York, NY, 2021; Impact, Schrödinger, LLC, New York, NY; Prime, Schrödinger, LLC, New York, NY, 2021.
5. Jacobson MP, Pincus DL, Rapp CS, et al. A hierarchical approach to all-atom protein loop prediction.Proteins 2004; 55:351-67. https://doi.org/10.1002/prot.10613
6. Jacobson MP, Friesner RA, Xiang Z, et al. On the role of the crystal environment in determining protein side-chain conformations. J Mol Biol 2002; 320:597-608. https://doi.org/10.1016/s0022-2836(02)00470-9
7. Schrödinger Release 2021-3: Prime, Schrödinger, LLC, New York, NY, 2021.
8. Davies MN, Toseland CP, Moss DS, et al. Benchmarking pK(a) prediction. BMC Biochem 2006; 7:18. https://doi.org/10.1186/1471-2091-7-18
9. Harder E, Damm W, Maple J, et al. OPLS3: A Force Field Providing Broad Coverage of Drug-like Small Molecules and Proteins. J Chem Theory Comput 2016; 12:281-96. https://doi.org/10.1021/acs.jctc.5b00864
10. Halgren T. Identifying and characterizing binding sites and assessing druggability. J Chem Inf Model 2009; 49:377-89. https://doi.org/10.1021/ci800324m
11. Halgren T. New method for fast and accurate binding-site identification and analysis. Chem Biol Drug Des.2007; 69:146-8. https://doi.org/10.1111/j.1747-0285.2007.00483.x
12. Schrödinger Release 2021-3: SiteMap, Schrödinger, LLC, New York, NY, 2021.
13. Friesner RA, Murphy RB, Repasky MP, et al. Extra precision glide: docking and scoring incorporating a model of hydrophobic enclosure for protein-ligand complexes. J Med Chem 2006; 49:6177-96. https://doi.org/10.1021/jm051256o
14. Halgren TA, Murphy RB, Friesner RA, et al. Glide: a new approach for rapid, accurate docking and scoring. 2. Enrichment factors in database screening. J Med Chem 2004; 47:1750-9. https://doi.org/10.1021/jm030644s.
15. Friesner RA, Banks JL, Murphy RB, et al. Glide: a new approach for rapid, accurate docking and scoring. 1. Method and assessment of docking accuracy. J Med Chem 2004; 47:1739-49. https://doi.org/10.1021/jm0306430

16. Schrödinger Release 2021-3: Glide, Schrödinger, LLC, New York, NY, 2021.

Table S1. Antioxidants information.

Product	Company	Cat#	Concentration
**Catalase from bovine liver**	Sigma-Aldrich	C1345-10G	1000 U/ml
**N-acetyl-L-cysteine (NAC)**	Sigma-Aldrich	A7250-25G	2 mM
**L-glutathione reduced (GSH)**	Sigma-Aldrich	G6013-5G	1 mM
**α-tocopherol (vitamin E)**	Sigma-Aldrich	258024-5G	20 µM
**Superoxide Dismutase (SOD)**	Sigma-Aldrich	S5395-15KU	200 U/ml

Table S2. Antibodies information.

Cell Signaling Technology				
**Antibody**	**Cat#**	**Lot#**	**Dilution**	**RRID**
**p-Akt (Ser473)**	9271	14	1:1,000	AB_329825
**Akt**	9272	22	1:1,000	AB_329827
**Bax**	2772	11	1:1,000	AB_10695870
**Bcl-xL**	2764	11	1:1,000	AB_2228008
**Bcl-2**	4223	6	1:1,000	AB_1903909
**BID**	2002	5	1:1,000	AB_10692485
**p-ERK (T202/Y204)**	9101	26	1:1,000	AB_331646
**ERK1/2**	9102	27	1:1,000	AB_330744
**p-SAPK/JNK (T183/Y185)**	9251	21	1:1,000	AB_331659
**Prdx1**	8499	1	1:1,000	AB_10950824
**p-p38 MAPK (T180/Y182)**	9216	27	1:2,000	AB_331296
**p38 MAPK**	9212	23	1:1,000	AB_330713
**p21 Waf1/Cip1**	2947	10	1:1,000	AB_823586
**p-Stat3 (S727)**	9134	20	1:1,000	AB_331589
**p-Stat3 (Y705)**	9131	30	1:1,000	AB_331586
**Stat3**	9139	8	1:2,000	AB_331757

**Wee1**	13084	1	1:4,000	AB_2713924
**p-Cdc2 (Y15)**	9111	11	1:8,000	AB_331460
**p27**	3686S	4	1:4,000	AB_2077850
**Santa Cruz Biotechnology**				
**Antibody**	**Cat#**	**Lot#**	**Dilution**	**RRID**
**Cyclin B1**	sc-245	F015	1:5,000	AB_627338
**GADPH**	sc-166574	I1013	1:10,000	AB_2107296
**JNK 1/3**	sc-474	A0109	1:1,000	AB_632383
**Mcl-1**	sc-12756	D2617	1:1,000	AB_627915
**β-actin**	sc-81178	J1116	1:2,000	AB_2223230
**anti-rabbit IgG-HRP**	sc-2357	A0318	1:5,000	AB_628497
**m-IgGκ BP-HRP**	sc-516102	L2017	1:5,000	AB_2687626
**Enzo Life Sciences**				
**Antibody**	**Cat#**	**Lot#**	**Dilution**	**RRID**
**Calnexin**	ADI-SPA-860-F	12301304	1:40,000	AB_11178981
**Caspase-3**	ADI-AAP-113	11021102	1:1,000	AB_10615972
**Caspase-8**	ADI-AAM-118-E	4041308	1:1,000	AB_2038943
**Caspase-9**	ADI-AAM-139-E	11221306	1:1,000	AB_2038946
**BioLegend**				
**Antibody**	**Cat#**	**Lot#**	**Dilution**	**RRID**
**p-H2AX (S139)**	613401	B182042	1:5,000	AB_315794
**BD Pharmingen**				
**Antibody**	**Cat#**	**Lot#**	**Dilution**	**RRID**
**PARP**	551024	38180	1:1,000	AB_394008
**Abcam**				
**Antibody**	**Cat#**	**Lot#**	**Dilution**	**RRID**
**Prdx-SO3**	ab16830	GR3294252-1	1:2,000	AB_443491
**BD Biosciences**				
**Antibody**	**Cat#**	**Lot#**	**Dilution**	**RRID**
**Cyclin A**	611268	5023928	1:6,000	AB_398796

EMD Millipore				
**Antibody**	**Cat#**	**Lot#**	**Dilution**	**RRID**
**p-Histone H3 (S10)**	06-570	3076467	1:5,000	AB_310177
**Bio-Rad**				
**Antibody**	**Cat#**	**Lot#**	**Dilution**	**RRID**
**anti-rabbit IgG-HRP**	170-6515	L1706515RevF	1:20,000	AB_11125142
**GE Healthcare Life Sciences**				
**Antibody**	**Cat#**	**Lot#**	**Dilution**	**RRID**
**anti-mouse IgG-HRP**	NA931	17179804	1:10,000	AB_772210

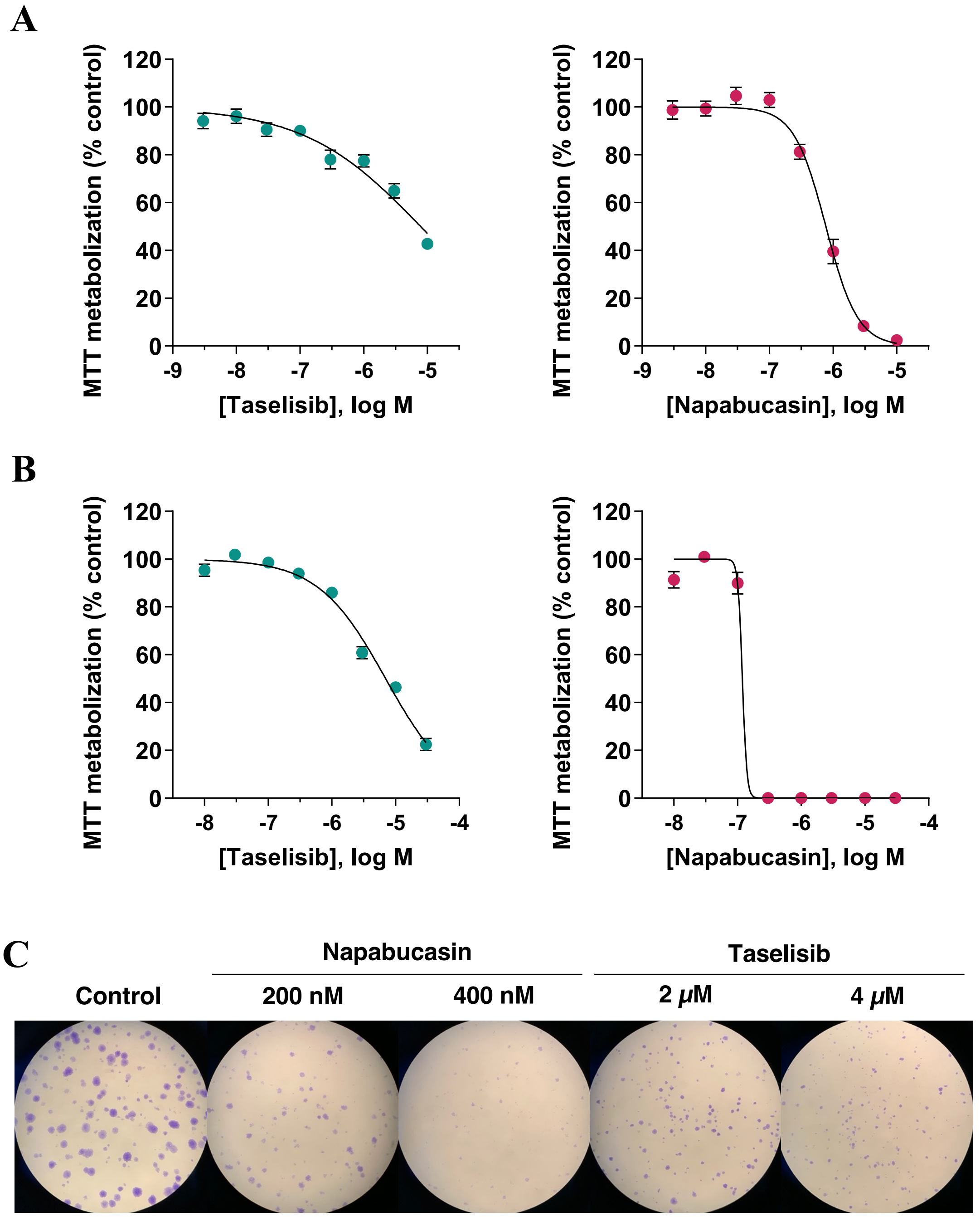

Figure S1. Effects of taselisib and napabucasin in TNBC cells. A-B, Effect of taselisib and napabucasin on cell viability, assessed by MTT metabolism at 48 h in MDA-MB-231 (A) and BT-549 cells (B) (n=2). **C,** Representative images of colony formation after 9 days of treatment with napabucasin and taselisib in MDA-MB-231 cells (n=2).

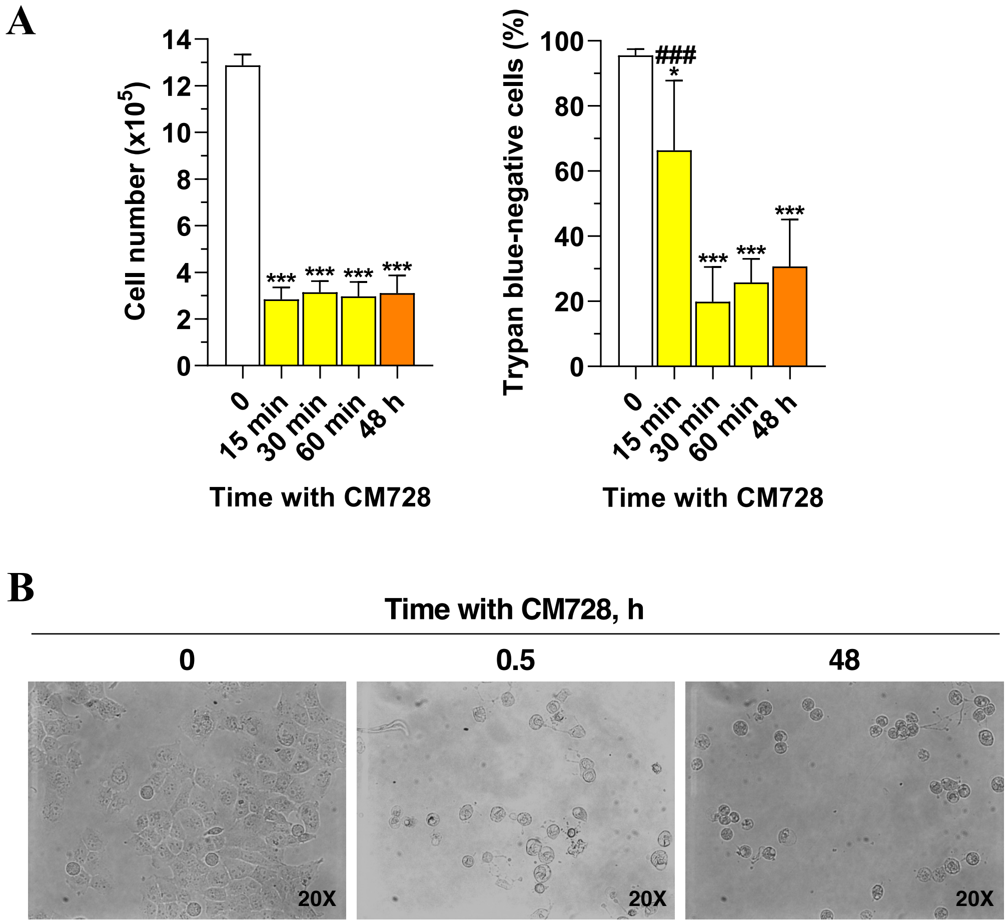

Figure S2. Effects of transient exposure to CM728 in MDA-MB-231 cells. A, Effect of pulse exposure of 1 µM CM728 on cell number (left) and the percentage of trypan blue-negative cells (right) at 48 h (n=2). Cell counting was performed using trypan blue staining. Statistical analyses were performed using one-way ANOVA and Dunnett's multiple comparison test. ***, P < 0.0001 *versus* control; $^{###}$, P < 0.0001 *versus* CM728 sustained-treated cells. **B,** Visualization of cells after a transient exposure to CM728 (1 µM). Images were obtained with an inverted phase-contrast microscope at 20x magnification.

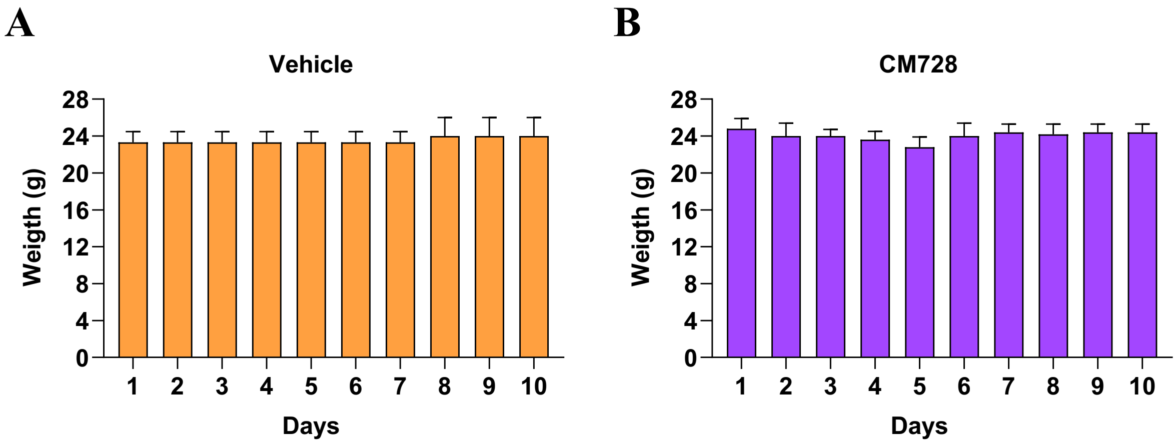

Figure S3. Evaluation of CM728 oral toxicity. A-B, Body weight of mice orally administered with vehicle (NMP/PEG400/0.1% methylcellulose in water for injection: 5/25/70) or CM728 (10 mg/kg) for 10 days.

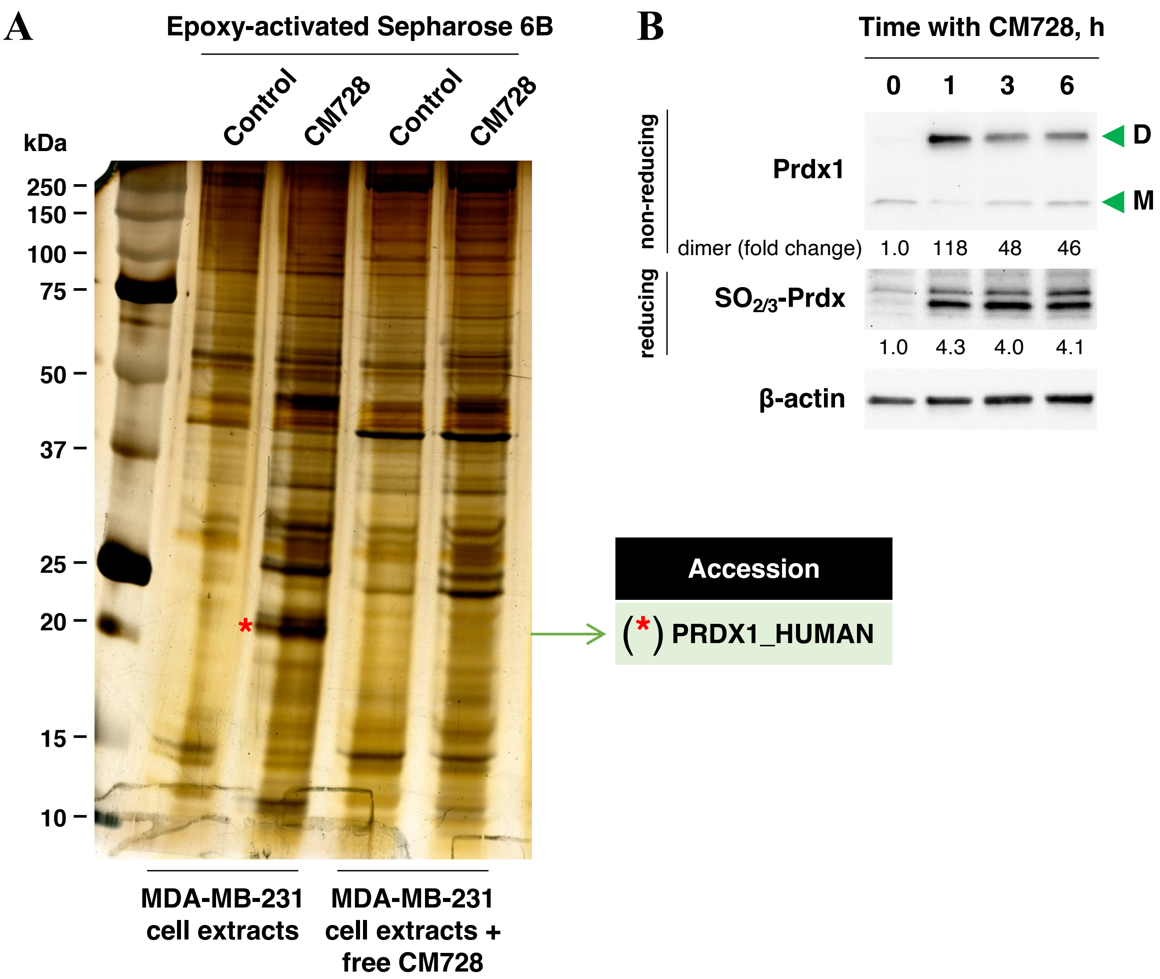

Figure S4. CM728 binds to Prdx1, inducing its dimerization and hyperoxidation. A, Identification of CM728-binding proteins in MDA-MB-231 lysates previously incubated with an excess of free CM728. Chemical proteomic approaches were carried out as specified in the Materials and Methods section. **B,** Immunoblot of the monomer (M) and dimer (D) forms of Prdx1, or the levels of hyperoxidized peroxiredoxin ($SO_{2/3}$-Prdx) in response to CM728 (1 µM) in MDA-MB-231 cells. β-actin was used as a loading control. Densitometry quantification from immunosignal values is shown below the bands. Values are relativized to the loading control. Representative images of two independent experiments are shown.

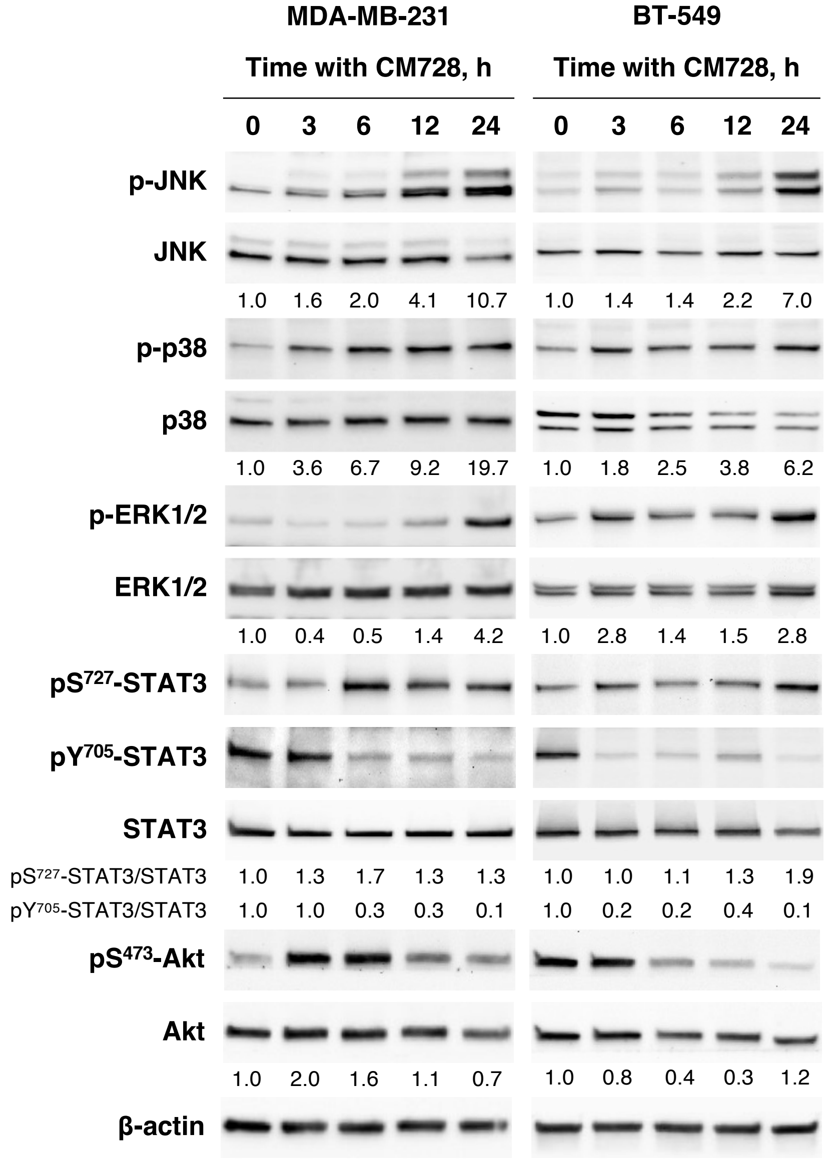

Figure S5. Phosphorylation of MAPKs, STAT3, and Akt in response to CM728 in TNBC cells. Effect of CM728 (1 μM) on JNK, p38 MAPK, ERK1/2, STAT3, and Akt phosphorylation in MDA-MB-231 (left) and BT-549 cells (right). β-actin was used as a loading control. Densitometric quantification from immunosignal values are shown below the bands. Phosphorylated forms of proteins are relativized to the total amount of the corresponding proteins. Values are relativized to the loading control. Representative images of two independent experiments are shown.

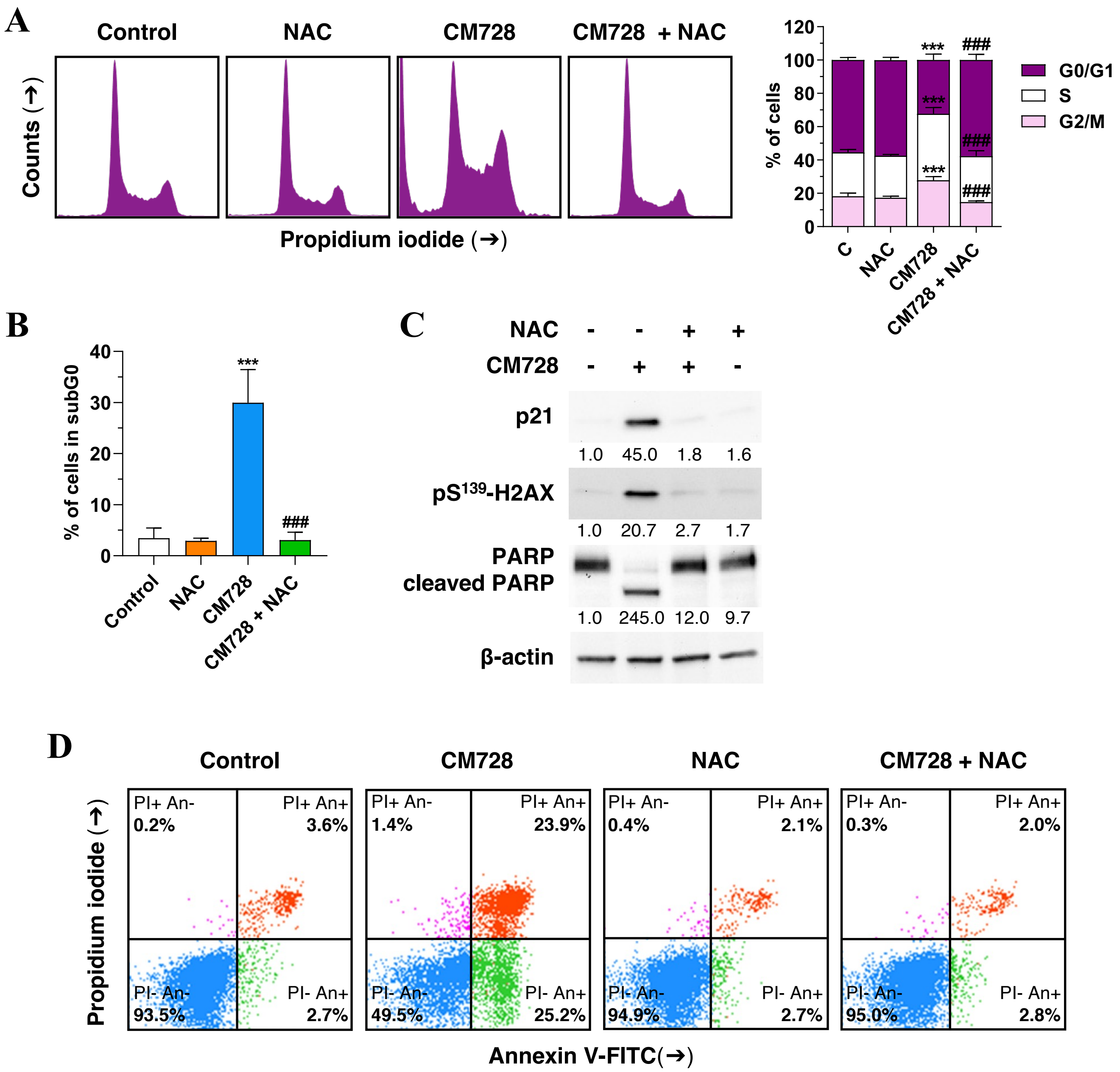

Figure S6. CM728-induced cell cycle arrest, DNA damage, and death was blocked by NAC. A-B, Cell cycle was analyzed by propidium iodide staining and flow cytometry in MDA-MB-231 cells that were preincubated with 2 mM NAC for 2 h and then treated with 1 µM CM728 for 48 h. **A,** Representative histogram (left), and percentage of cells in each cycle phase (right); and, **B,** percentage of subG0 population are shown (n=2) **C,** Analysis of p21, pS^{139}-H2AX and PARP in MDA-MB-231 cells that were preincubated with 2 mM NAC for 2 h and then treated with 1 µM CM728 for 24 h. β-actin was used as loading control. Densitometry quantification from immunosignal values relativized to the loading control is shown below the bands. Representative images of two independent experiments are shown. **D,** Effect of NAC on the CM728-induced apoptosis in MDA-MB-231 cells. The effect of 2 mM NAC (2 h of preincubation) in the absence and presence of 1 µM CM728 (48 h of incubation) was studied by flow cytometry after Annexin V/PI double staining (n=2). Representative images of two independent experiments are shown. PI: propidium iodide; An: annexin V. Statistical analyses were performed using two-way ANOVA followed by Tukey's post-hoc test (A) or using one-way ANOVA, and Dunnett's multiple comparison test (B). ***, $P < 0.0001$ *versus* control; ###, $P < 0.0001$ *versus* CM728-treated cells.

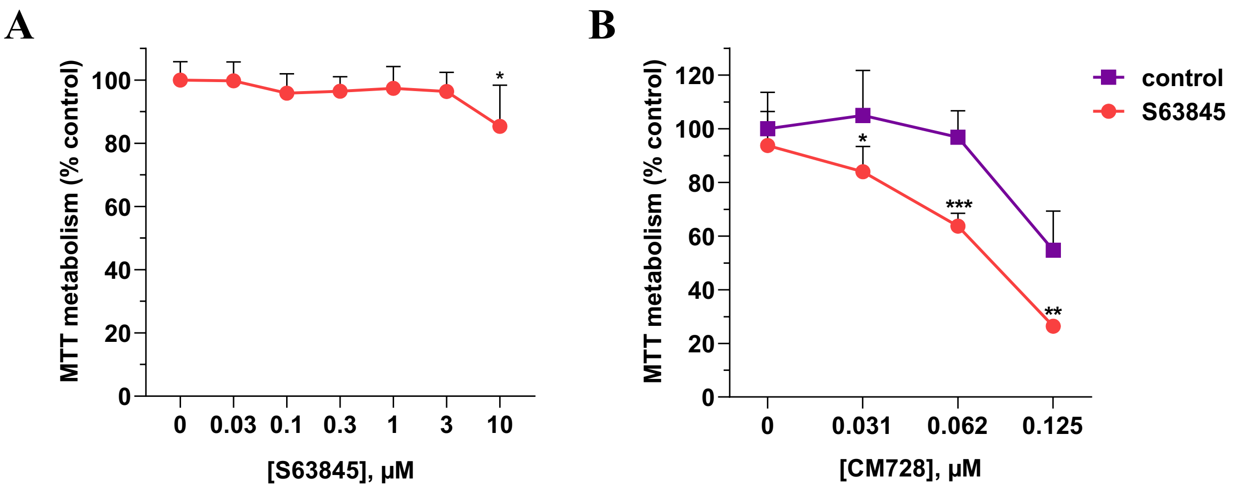

Figure S7. CM728 antitumor activity was enhanced by S63845. A, The cytotoxicity of S63845 was measured by MTT assays after 48 h of incubation in MDA-MB-231 cells (n=2). Statistical analyses were performed with one-way ANOVA, and Dunnett's multiple comparisons test. *, $P < 0.05$ *versus* cells treated with vehicle. **B,** The effect of the indicated concentrations of CM728 alone or in combination with S63845 (0.5 μM) was assessed by MTT metabolism after 72 h of treatment in MDA-MB-231 cells (n=2). Statistical analyses were performed with one-way ANOVA, and Dunnett's multiple comparisons test. *, $P < 0.05$ *versus* cells treated with CM728 alone; **, $P < 0.001$ *versus* cells treated with CM728 alone; ***, $P < 0.0001$ *versus* cells treated with CM728 alone.
